# Halide Perovskite–Chalcohalide Nanocrystal Heterostructures as a Platform for the Synthesis and Investigation of the CsPbCl_3_–CsPbI_3_ Epitaxial Interface

**DOI:** 10.1002/adma.202512502

**Published:** 2025-11-06

**Authors:** Nikolaos Livakas, Irina Skvortsova, Juliette Zito, Yurii P. Ivanov, Aswin Asaithambi, Andrea Toma, Annick De Backer, Muhammad Imran, Sandra Van Aert, Giorgio Divitini, Ivan Infante, Sara Bals, Liberato Manna

**Affiliations:** ^1^ Nanochemistry Istituto Italiano di Tecnologia Via Morego 30 Genova 16163 Italy; ^2^ Electron Spectroscopy and Nanoscopy Istituto Italiano di Tecnologia Via Morego 30 Genova 16163 Italy; ^3^ Clean Room Facility Istituto Italiano di Tecnologia Via Morego 30 Genova 16163 Italy; ^4^ Electron Microscopy for Materials Science (EMAT) University of Antwerp Antwerp 2020 Belgium; ^5^ NANOlab Center of Excellence University of Antwerp Antwerp 2020 Belgium; ^6^ BCMaterials, Basque Center for Materials, Applications, and Nanostructures UPV/EHU Science Park Leioa 48940 Spain; ^7^ Department of Electrical and Computer Engineering University of Toronto 10 King's College Road Toronto ON M5S 3G4 Canada

**Keywords:** halide exchange, halide perovskites, heterostructures, interfaces, nanocrystals

## Abstract

Halide exchange in lead‐based halide perovskites has been studied extensively. While mixed Cl/Br or Br/I alloy compositions can be formed with no miscibility gaps, this is precluded for mixed Cl/I compositions, due to the large difference in Cl^−^ and I^−^ ionic radii. Here, perovskite‐chalcohalide CsPbCl_3_–Pb_4_S_3_Cl_2_ nanocrystal heterostructures are exploited to study the Cl→I exchange and to isolate new types of intermediate structures. The epitaxial interface between the Pb_4_S_3_Cl_2_ chalcohalide and the CsPbCl_3_ perovskite significantly influences the intermediate stages of halide exchange in the perovskite domain, leading to coexisting CsPbCl_3_ and CsPbI_3_ domains, thereby delivering segmented CsPbI_3_–CsPbCl_3_–Pb_4_S_3_Cl_2_, energetically favorable heterostructures, with partial I‐alloying of the CsPbCl_3_ domain and at the perovskite–chalcohalide interface. The I:CsPbCl_3_ domain between CsPbI_3_ and Pb_4_S_3_Cl_2_ enables a gradual lattice expansion across the heterostructure. This design accommodates interfacial strain, with a 5.6% mismatch at the CsPbCl_3_–CsPbI_3_ interface and a 3.4% mismatch at the perovskite–chalcohalide interface. Full halide exchange leads to CsPbI_3_–Pb_4_S_3_Cl_2_ heterostructures. Both in intermediate and fully exchanged heterostructures, the CsPbI_3_ domain is emissive. In the intermediate structures, the band alignment between the two perovskite domains is type‐I, with the carriers photogenerated in the CsPbCl_3_ domain quickly transferring to the CsPbI_3_ domain, where they can recombine radiatively.

## Graphical abstract

In this work, perovskite–chalcohalide CsPbCl_3_–Pb_4_S_3_Cl_2_ heterostructures are exploited to study the Cl→I exchange reaction and to isolate new types of intermediate structures, in particular emissive CsPbI_3_–CsPbCl_3_–Pb_4_S_3_Cl_2_ heterostructures, with partial I‐alloying of the CsPbCl_3_ domain and at the perovskite–chalcohalide interface, having a 5.6% lattice mismatch at the CsPbCl_3_–CsPbI_3_ interface and a residual 3.4% mismatch at the perovskite–chalcohalide interface.

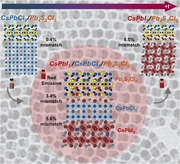

## 1 Introduction

**Scheme 1:**
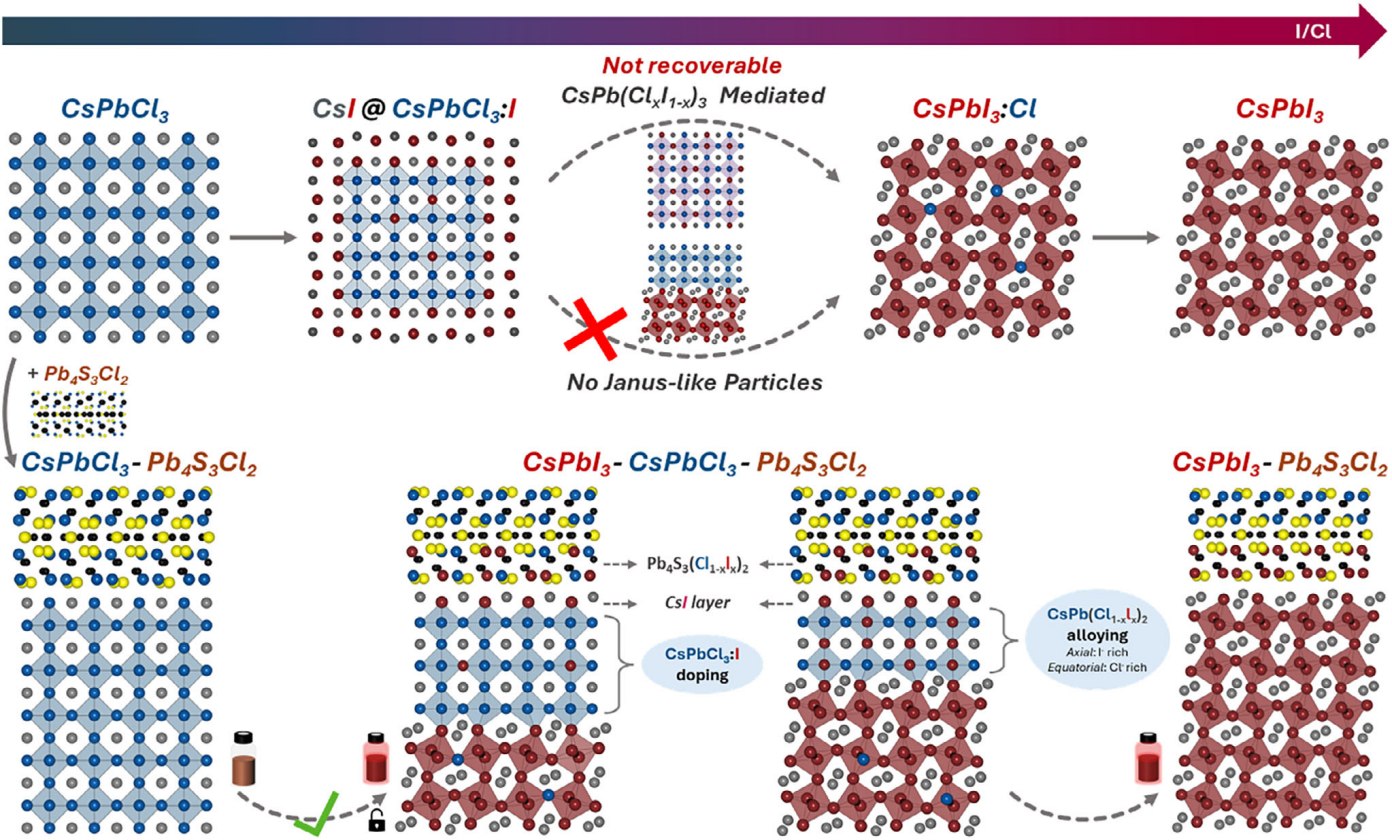
Schematic representation of the Cl→I exchange on both free‐standing CsPbCl_3_ NCs and CsPbCl_3_–Pb_4_S_3_Cl_2_ heterostructure NCs. As discussed in a previous work of ours,^[^ ^13^ ^]^ anion exchange in the case of free‐standing CsPbCl_3_ NCs (top row of panels) proceeds through the initial formation of CsPbCl_3_ NCs alloyed with ≈5% iodine and covered with an exchanged CsI layer. As soon as the iodine concentration reaches its solubility limit in the CsPbCl_3_ phase, the CsPbCl_3_:I NCs rapidly turn into CsPbI_3_:Cl NCs (with ≈4% Cl^−^ in CsPbI_3_), jumping over the miscibility gap. Pure CsPbI_3_ NCs are obtained after expelling the residual Cl^−^ ions from the lattice. No other intermediate CsPb(Cl_1−x_I_x_)_3_ alloy compositions nor phase‐segregated CsPbI_3_–CsPbCl_3_ intermediates could be recovered.^[^ ^13^ ^]^ In the case of the CsPbCl_3_–Pb_4_S_3_Cl_2_ NC heterostructures of this work, the same reaction follows a different pathway (bottom row of panels). The exchange starts with I^−^ ions replacing Cl^−^ at the perovskite–chalcohalide interface and at the bottom of the CsPbCl_3_ domain, converting part of it into CsPbI_3_ and forming an epitaxial CsPbCl_3_–CsPbI_3_ interface. As the reaction progresses, most of the perovskite domain transforms into CsPbI_3_, reducing the CsPbCl_3_ domain to a few atomic rows in projection. At this late stage of the exchange, the CsPbCl_3_ domain exhibits considerable alloying with I^−^ ions. Also, these ions are preferentially distributed along the vertical direction. This process effectively leads to segmented CsPbI_3_–CsPbCl_3_–Pb_4_S_3_Cl_2_ red‐emitting heterostructures. With enough I^−^, fully exchanged CsPbI_3_‐chalcohalide heterostructure NCs are obtained.

Lead halide perovskite nanocrystals (NCs) have gathered significant attention due to their appealing optical properties and potential for various applications.^[^ ^1^, ^2^, ^3^, ^4^, ^5^, ^6^ ^]^ Soon after their synthesis was developed,^[^ ^7^, ^8^ ^]^ various studies addressed their reactivity.^[^ ^9^ ^]^ In this context, halide exchange reactions are particularly important for these materials as they can be conveniently harnessed to fine‐tune the optical bandgap and achieve any desired emission color, from the blue to the red region of the visible spectrum.^[^ ^10^, ^11^ ^]^ In the past, almost all studies were focused on the Cl ↔ Br and Br ↔ I reactions, and it was assessed that all intermediate Cl‐Br and Br‐I alloy compositions can be prepared. On the other hand, the direct Cl ↔ I exchange was much less understood and studied, given the large difference in ionic radii between Cl^−^ and I^−^ ions.^[^ ^10^, ^11^, ^12^ ^]^ Hence, it was unclear what types of intermediate structures could be formed during such exchange reactions. In a recent work of ours,^[^ ^13^ ^]^ we explored the direct exchange of Cl^−^ with I^−^ ions in CsPbCl_3_ NCs and discovered that the intermediate products of this reaction consist of a mixture of pristine (i.e. “unexchanged”) CsPbCl_3_ NCs and fully exchanged (i.e. CsPbI_3_) NCs. Based on X‐ray diffraction (XRD) analysis, both types of NCs had very limited alloy compositions (≈6% I^−^ in CsPbCl_3_, and ≈4% Cl^−^ in CsPbI_3_), at the edges of the miscibility gap. Neither NCs with CsPb(Cl_1−x_I_x_)_3_ homogeneous alloy compositions nor with heteroepitaxial CsPbI_3_–CsPbCl_3_ interfaces could be isolated in those intermediate exchanged samples. The only alternative way for iodine to be added to the CsPbCl_3_ NCs was to form a CsI surface layer. These results are summarized in **Scheme** **1**, top row of panels.

While these results are in line with structural/thermodynamic predictions, our further aim was to investigate whether the introduction of additional structural constraints on a starting CsPbCl_3_ NC could influence the outcome of such halide exchange reaction, especially at the intermediate stages. One possible structural constraint is represented by the epitaxial interface that a CsPbCl_3_ NC already shares with another material, as is the case for the perovskite–chalcohalide CsPbCl_3_–Pb_4_S_3_Cl_2_ NC heterostructures that were recently synthesized by us.^[^ ^14^, ^15^ ^]^ In the present work, we therefore investigated the Cl→I exchange reaction in such CsPbCl_3_–Pb_4_S_3_Cl_2_ NC heterostructures, and we were able to reveal distinct differences in the partial exchange case compared to free‐standing CsPbCl_3_ NCs (Scheme 1). At intermediate exchange stages, the CsPbCl_3_ domain farthest from the perovskite–chalcohalide interface undergoes complete anion exchange with I^−^ ions, forming a heteroepitaxial CsPbCl_3_–CsPbI_3_ interface with the remaining portion of the CsPbCl_3_ domain. Concurrently, I^−^ ions penetrate and partially replace Cl^−^ ions across three atomic layers at the perovskite–chalcohalide interface: the terminal CsCl monolayer of the perovskite domain converts to CsI, along with the shared atomic layer between the CsPbCl_3_ and Pb_4_S_3_Cl_2_ domains, and the first layer of the chalcohalide domain, forming a Pb_4_S_3_(Cl_1−x_I_x_)_2_ structure. Interestingly, as the exchange progresses and the remaining CsPbCl_3_ domain is reduced to a few atomic rows, it starts alloying with iodine, with I^−^ ions preferentially settling in rows perpendicular to the CsPbCl_3_–CsPbI_3_ and perovskite–chalcohalide interfaces (Scheme 1, bottom row of panels). The alloyed I:CsPbCl_3_ domain, with a lattice parameter of 5.93 Å, which is intermediate between those of CsPbI_3_ (6.26 Å) and Pb_4_S_3_Cl_2_ (5.73 Å), enables a gradual lattice expansion that produces a graded structure, with ≈5.6% lattice mismatch at the CsPbCl_3_–CsPbI_3_ interface and ≈3.4% residual mismatch at the perovskite–chalcohalide interface. Density functional theory (DFT) calculations on fully atomistic models supported these observations. All these substitutions collectively lead to energetically favorable configurations during the various stages of the exchange process: first, initiating the Cl→I exchange at the bottom of the perovskite domain represents an energetic minimum among possible Cl^−^/I^−^ distributions, thus favoring the segmented CsPbI_3_–CsPbCl_3_–Pb_4_S_3_Cl_2_ heterostructure. This energetic preference suggests that the region farthest from the interface, with fewer structural constraints, enables complete Cl→I exchange. Likewise, the vertical ordering of I^−^ ions in the remaining CsPbCl_3_ domain at the later stage of exchange corresponds to an energetically favorable arrangement. Finally, provided enough I^−^ ions are present, the exchange can be brought to completion, thus generating CsPbI_3_‐chalcohalide NCs. Remarkably, the partial (or full) exchange converts the initially non‐emissive, type‐I CsPbCl_3_–Pb_4_S_3_Cl_2_ heterostructures into emissive ones. In particular, the intermediate CsPbI_3_–CsPbCl_3‐_‐Pb_4_S_3_Cl_2_ heterostructure presents band alignments such that emission from the CsPbCl_3_ domain is still quenched, while emission from the CsPbI_3_ domain is possible. Transient absorption measurements evidenced a faster decay of the CsPbCl_3_ exciton when CsPbCl_3_ is interfaced to both CsPbI_3_ and Pb_4_S_3_Cl_2_ domains compared to the pristine CsPbCl_3_–Pb_4_S_3_Cl_2_ heterostructure, indicating the formation of a type‐I alignment between the CsPbCl_3_ and CsPbI_3_ domains. In such configuration, the photoexcited carriers in the CsPbCl_3_ can be transferred to either the chalcohalide or the CsPbI_3_ domains, and when they are transferred to the CsPbI_3_ domain, they recombine radiatively.

Overall, our work demonstrates that NC heterostructures enable the formation of epitaxial interfaces and alloy compositions (hence, new phases) that have not been reported so far neither in bulk solids nor in isolated nanocrystals.

## 2 Results and Discussion

**Figure 1:**
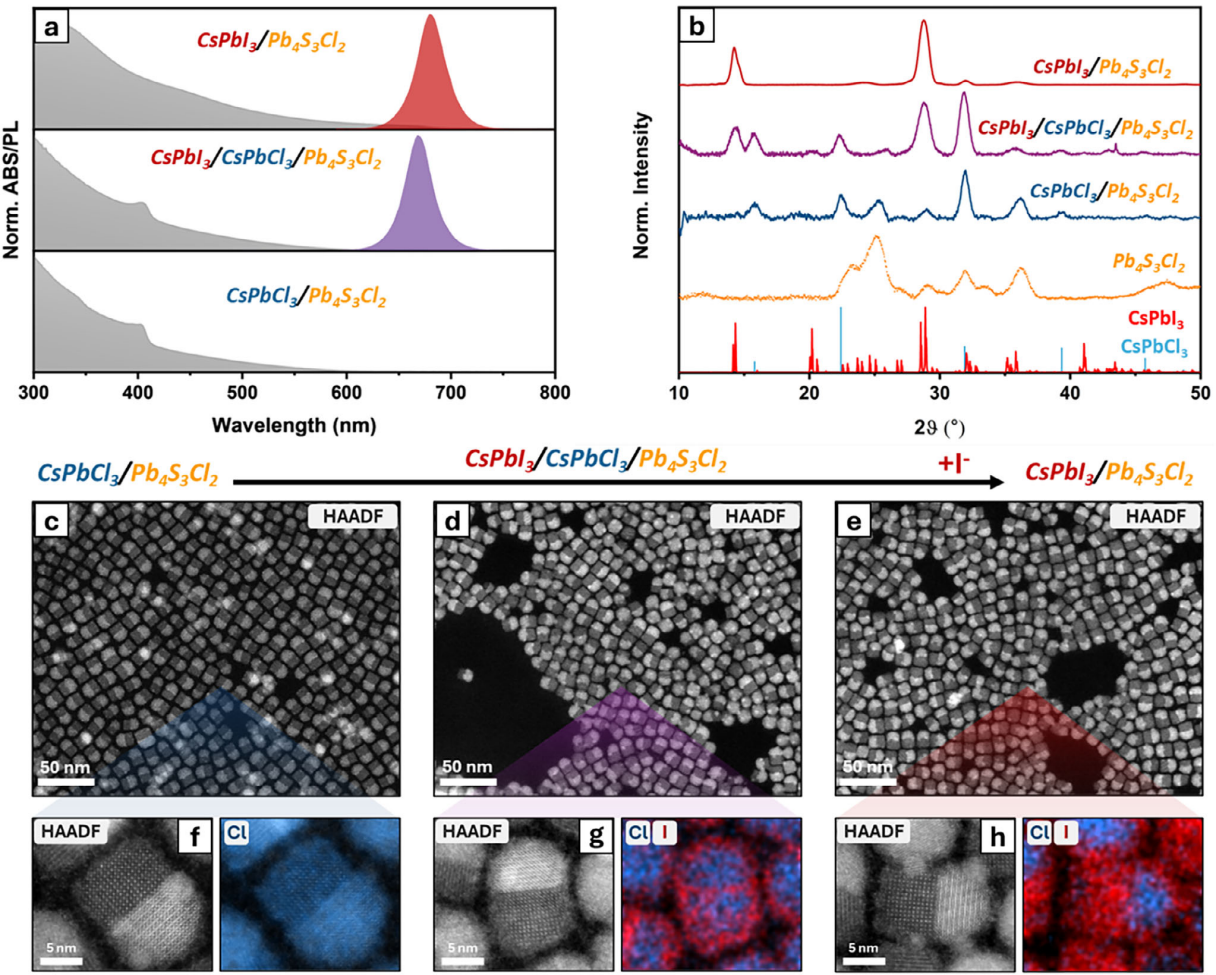
Steady‐state optical, XRD, and electron microscopy analyses of the initial, intermediate and final samples for the Cl→I exchange reaction on CsPbCl_3_–Pb_4_S_3_Cl_2_ NC heterostructures. (a) Optical absorption (gray) and PL (colored) spectra of the pristine, partially exchanged (X_cl_ = 52%) and fully exchanged (X_cl_ = 5%) heterostructures (X_cl_, defined as: X_cl_ = [Cl]/([Cl]+[I])*100, is calculated from STEM‐EDX data). XRD patterns (b) and HAADF‐STEM images (c–e) along with the corresponding STEM‐EDX elemental maps (f–h). In (b), the XRD pattern of a powder sample of separately synthesized Pb_4_S_3_Cl_2_ NCs is also shown for comparison.

The CsPbCl_3_/Pb_4_S_3_Cl_2_ NC heterostructures studied in this work were prepared following the procedure published by our group in a previous work.^[^ ^14^ ^]^ Briefly, the synthesis consists of a two‐step approach: the first step involves the preparation of CsPbCl_3_ clusters at a relatively low temperature (50 °C) over a long reaction time (30 min). The second step involves consecutive injections of Pb‐oleate, dodecanethiol, CsPbCl_3_ clusters, and elemental sulfur (dissolved in octadecene) into pre‐degassed octadecene at 200 °C under inert atmosphere (see Experimental Section). The prepared CsPbCl_3_/Pb_4_S_3_Cl_2_ NCs were then subjected to I^−^ exchange by employing PbI_2_ (in the presence of oleylamine and oleic acid) as a halide source. **Figure** **1** reports optical absorption (ABS) and photoluminescence (PL) spectra, along with X‐ray diffraction (XRD) patterns of the initial and I^−^ exchanged samples. The optical spectra (Figure 1a) and the XRD patterns (Figure 1b) demonstrate the progressive, partial to complete halide exchange, depending on the amount of PbI_2_ precursor added. Consistent with our prior findings on isolated CsPbCl_3_ NCs,^[^ ^13^ ^]^ PbI_2_ incorporation induces the formation of the CsPbI_3_ phase alongside the initial CsPbCl_3_ phase (and the Pb_4_S_3_Cl_2_ here). This new CsPbI_3_ phase is responsible for the PL emission in the red spectral region, while no emission is seen in the initial heterostructures. With increasing iodine source, the PL peak red shifts until the reaction reaches the stage of fully exchanged CsPbI_3_/Pb_4_S_3_Cl_2_ NCs (Figure 1a,b).

High‐angle annular dark‐field scanning transmission electron microscopy (HAADF‐STEM) analysis indicates that halide exchange does not alter the overall NCs’ morphology (Figure 1c–e). However, energy dispersive X‐ray spectroscopy maps acquired in STEM mode (STEM‐EDX, see Figure 1f–h; Figure S1, Supporting Information) reveal compositional changes. Elemental analysis of a single partially exchanged NC shows evidence of a significant presence of iodine alongside chlorine in the perovskite domain, suggesting the coexistence of CsPbCl_3_ and CsPbI_3_ phases within the same particle (Figure 1g; Figure S1, Supporting Information). Iodine species are also detected at the perovskite–chalcohalide interface and surrounding the chalcohalide domain. Interestingly, HAADF‐STEM images of the NCs that appear to be in the early stages of the reaction indicate the formation of a CsI passivation layer on the CsPbCl_3_ surface (Figure S2, Supporting Information), similarly to what was observed in isolated CsPbCl_3_ NCs.^[^ ^13^ ^]^ The iodine incorporation is also observed at the perovskite–chalcohalide interface (Figure S2, Supporting Information, red arrows). In fully exchanged CsPbI_3_/Pb_4_S_3_Cl_2_ NCs, iodine remains present around the chalcohalide domain (Figure 1h). Notably, the chalcohalide domain never gets exchanged beyond its surface region, indicating a very low diffusivity of the halide ions in such material.

**Figure 2:**
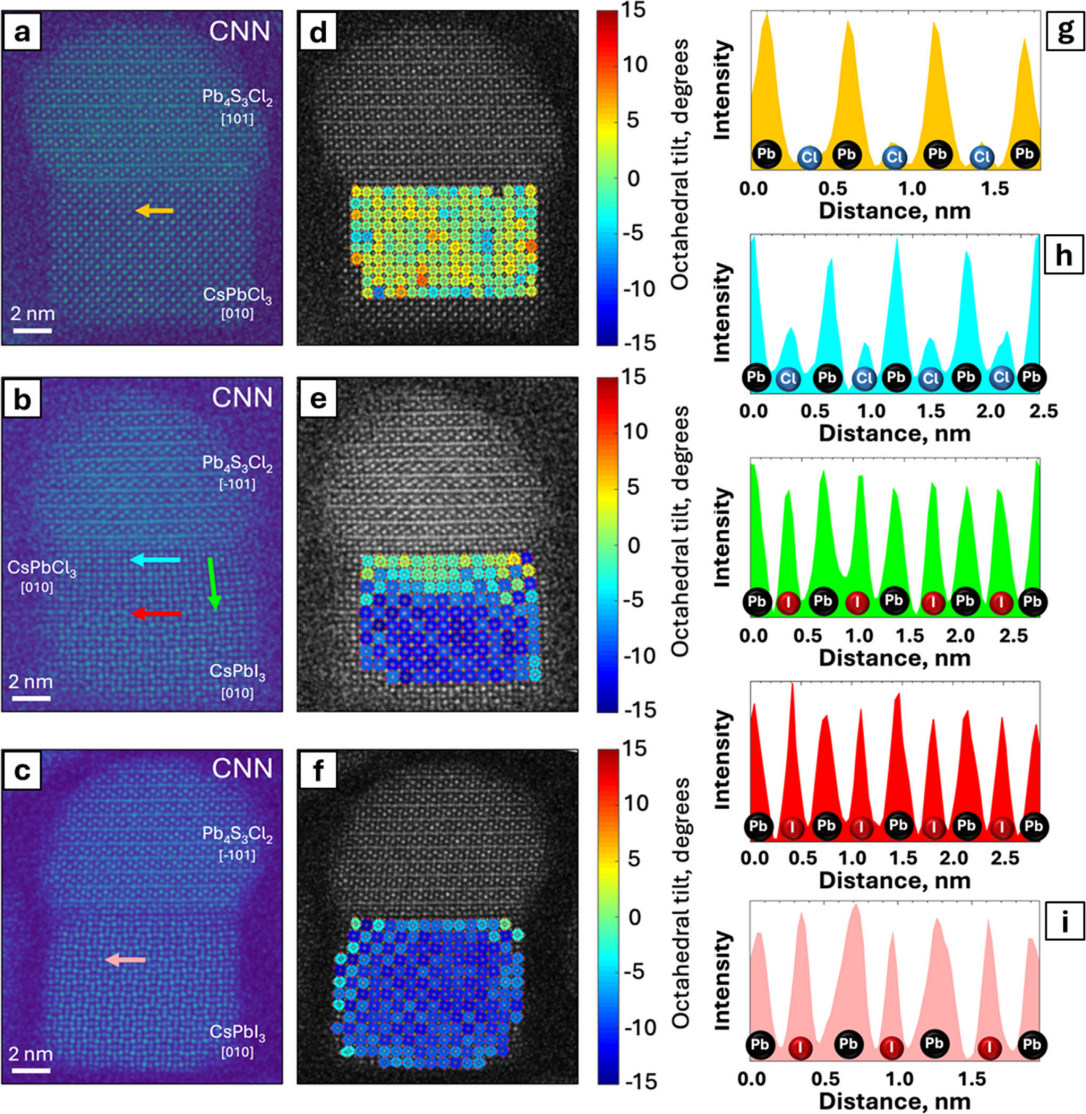
Phase images obtained from CNN reconstruction of 4D‐STEM dataset of the starting CsPbCl_3_–Pb_4_S_3_Cl_2_ (a), partially exchanged CsPbCl_3_–CsPbI_3_–Pb_4_S_3_Cl_2_ (b) and fully exchanged CsPbI_3_–Pb_4_S_3_Cl_2_ (c) heterostructures. The color‐coded octahedral tilt mapping (d,e,f) was calculated for (a,b,c). Intensity profiles (g,h,i) correspond to the arrows in (a,b,c) where Pb+X columns are labelled as Pb and pure X columns are labelled as Cl or I. Although some degree of iodine may be present in chlorine columns and vice versa, the labels denote the predominant element in each column. The octahedral tilt analysis estimates the averaged angle between the lattice vectors and the vectors corresponding to the direction of PbX_6_ octahedra, thus, reflecting the Pb‐Pb‐X angle.

HAADF‐STEM imaging typically requires relatively high electron doses to achieve a sufficient signal‐to‐noise ratio. It is, however, well‐known that these doses can induce beam damage and ion diffusion within the perovskite structure, leading, for example, to the formation of Pb clusters on the NC surface.^[^ ^16^, ^17^, ^18^ ^]^ To overcome this challenge and to further investigate the Cl→I exchange on heterostructures, we employed 4D‐STEM with an event‐driven direct electron detector. In earlier works, a convolutional neural network (CNN) was trained to output the phase image from such datasets.^[^ ^19^, ^20^ ^]^ A main advantage of this approach is that the required electron dose could be reduced from >1000 e^−^/Å^2^, as in the case of HAADF‐STEM, to 300 e^−^/Å^2^, as for CNN images in the current work, which is important for the investigation of local changes in structure and composition without the influence of the electron beam. As illustrated in **Figure** **2**a–c, there are only minor changes in the shape of the exchanged heterostructures in comparison to the initial CsPbCl_3_/Pb_4_S_3_Cl_2_ NCs. Specifically, in partially exchanged NCs, the top region of the perovskite domain, connected to the chalcohalide domain, remains compressed in the direction parallel to the interface (Figure 2b). This morphological feature might indicate the presence of structurally different regions within the perovskite domain. To assess this hypothesis, we performed octahedral tilt measurements based on the CNN images (Figure 2d–f, see details in the experimental section). As expected, the octahedral tilt for the α‐CsPbCl_3_ domain in the initial heterostructure is close to zero (Figure 2d), whereas for the fully exchanged γ‐CsPbI_3_ domain in the final exchanged heterostructure the octahedral tilt values reach up to 14 degrees (Figure 2f). Interestingly, for the partially exchanged case (Figure 2e), we can distinguish two different areas with tilt distributions fluctuating at ≈0 and 14 degrees, which effectively demonstrates the coexistence of CsPbCl_3_–CsPbI_3_ domains sharing an epitaxial interface. Such distribution of phases in partially exchanged NCs reveals unique Cl→I exchange dynamics in the heterostructures, different from the free‐standing NCs observed by us in our previous work.^[^ ^13^ ^]^

Notably, in the CNN‐reconstructed images, the intensities of the atomic columns are more sensitive to light elements than in HAADF‐STEM images.^[^ ^20^ ^]^ Thus, this approach enables direct visualization of both chlorine and iodine atomic columns (Figure 2a,c), such that they can be reliably distinguished (Figure 2g,i). Therefore, in addition to structural information, the CNN‐reconstructed images provide qualitative insight into the relative composition of halide atomic columns based on their intensity variations. In the following lines, we focus on a single heterostructure at a relatively advanced stage of exchange as this reveals a wide richness of features. From the intensity profiles (Figure 2h; Figures S3 and S4, Supporting Information) derived from the CNN image of this heterostructure we can draw several conclusions. The profiles confirm the coexistence of CsPbCl_3_–CsPbI_3_ domains, as illustrated by the cyan and red intensity profiles in Figure 2h as well as additional supporting profiles, provided in Figure S3 (Supporting Information). As seen in the cyan profile in Figure 2h, the intensity peaks located between the Pb + X columns, corresponding to pure halide columns, are substantially lower than those observed in the red profile. The peak heights in the cyan profile resemble those of Cl columns (Figure 2g), whereas the red profile aligns more closely with the intensity of I columns (Figure 2i). Interestingly, the CsPbCl_3_ domain appears to be alloyed with iodine, with the I^−^ ions concentrated primarily along the vertical direction within the CsPbCl_3_ lattice (green profile in Figure 2h; Figure S4, Supporting Information). In contrast, we also observed heterostructures with slightly larger CsPbCl_3_ domains (evidently captured at an earlier stage of halide exchange) showing no evidence of iodine incorporation, as confirmed by intensity profiles in both vertical and horizontal directions (Figure S5, Supporting Information). The comparison between these two heterostructures (Figure 2b; Figure S5, Supporting Information) provides insight into the exchange dynamics: initially, distinct domains of pure CsPbCl_3_ and CsPbI_3_ within a single heterostructure share an epitaxial interface. As the halide exchange progresses, this is followed by a reduction in size of the CsPbCl_3_ domain and by its substantial alloying with iodide ions, the latter replacing chloride ions predominantly along directions vertical to the CsPbCl_3_–CsPbI_3_ interface, with the cubic crystal structure in the CsPbCl_3_ region being preserved.

The lattice parameter analysis for the partially exchanged heterostructures (Figure S6, Supporting Information) reveals lattice expansion when moving away from the perovskite–chalcohalide interface, supporting the structural distinction between the CsPbCl_3_ (cubic phase, *Pm‐3m*) and CsPbI_3_ (gamma phase, *Pnma*) domains, as shown earlier with the octahedral tilt measurements (Figure 2e). A similar intensity and structural investigation was performed on the partially exchanged heterostructures where the CsPbI_3_ domain is aligned along the [101] direction (Figure S7, Supporting Information), and the results are in good agreement with the conclusions presented above, proving the presence of two distinct structural subdomains (Figures S7d,e and S8, Supporting Information) within the perovskite region as well as significant alloying of the CsPbCl*_3_* domain with iodine (Figure S7b, Supporting Information).

**Figure 3:**
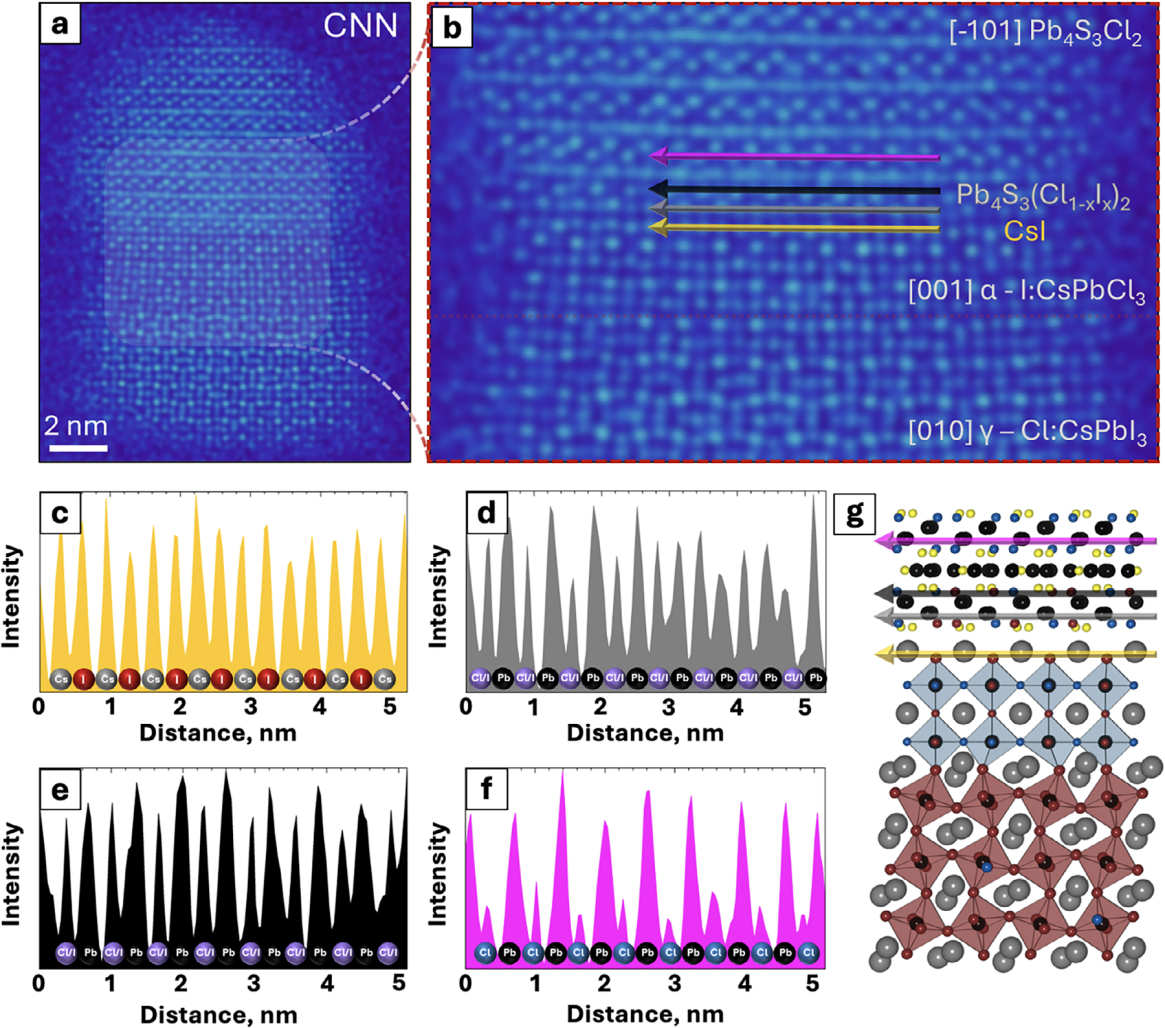
(a) Phase image obtained from CNN reconstruction of a 4D‐STEM dataset of the partially exchanged CsPbI_3_–CsPbCl_3_–Pb_4_S_3_Cl_2_ NC heterostructure presented in Figure 2b. (b) Enlarged area from the same high‐resolution CNN image. The arrows correspond to the intensity profiles represented in panels (c–f) showing three atomic layers at the CsPbCl_3_–Pb_4_S_3_Cl_2_ interface exchanged with I^−^ (c,d,e). (g) Reconstructed model corresponding to the multidomain structure presented in panel (a).

Intensity profile analyses of CNN images also offer qualitative insights concerning the composition near the perovskite–chalcohalide interface (**Figure** **3**). A CsI layer separates the perovskite and chalcohalide domains (Figure 3c). Such iodine segregation at the perovskite–chalcohalide interface was also observed in the STEM‐EDX maps of Figure 1g,h. The atomic layers in the chalcohalide domain immediately adjacent to the interface are also alloyed with iodine, thus leading to a Pb_4_S_3_(Cl_1−x_I_x_)_2_ composition there (Figure 3d,e). Further beyond these layers, the peak heights of the halide columns decrease (Figure 3f), suggesting that deeper regions in the chalcohalide domain preserve their pristine Pb*_4_* S*_3_* Cl*_2_ *composition.

To rationalize the difference in behavior of heterostructures vs free‐standing CsPbCl_3_ nanocubes upon halide exchange, we further investigated the epitaxial relation between chalcohalide and perovskite phases. Although CsPbCl_3_ crystallizes in the cubic *Pm‐3m* space group and Pb_4_S_3_Cl_2_ adopts an orthorhombic *Pnma* structure, their lattice parameters are sufficiently close to allow epitaxial matching. On the other hand, CsPbI_3_ crystallizes in the *Pnma* space group, the same as Pb_4_S_3_Cl_2_. The lattice mismatch was calculated based on the average lattice parameters measured from HAADF‐STEM images (Figure S9, Supporting Information) for each phase through the following expression:

(1) $$\mathrm{lattice}\;\mathrm{mismatch}\;=\left(\frac{a_{perovskite}-a_{chalcohalide}}{a_{perovskite}}\cdot 100\%\right)$$

For the initial CsPbCl_3_–Pb_4_S_3_Cl_2_ heterostructures, the lattice mismatch is almost negligible (≈0.4%, Figure S9a,b,c, Supporting Information). In the case of partially exchanged heterostructures, the presence of an alloyed I:CsPbCl_3_ domain between CsPbI_3_ and Pb_4_S_3_Cl_2_ allows a gradual expansion of the lattice parameters (Figure S9d,e,f, Supporting Information). The mismatch at the CsPbCl_3_–CsPbI_3_ interface is ≈5.6%, whereas the mismatch at the perovskite–chalcohalide interface is ≈3.4% (Figure S9e,f, Supporting Information). However, this latter value can be expected to already include some compensation due to iodide incorporation into the chalcohalide region close to the interface (Figure 3d,e; Figure S9f, Supporting Information), which has a Pb_4_S_3_(Cl_1−x_I_x_)_2_ composition. The observed overall gradient in lattice mismatch most likely favors the formation of CsPbI_3_–CsPbCl_3_–Pb_4_S_3_Cl_2_ heterostructures and makes it possible to capture the CsPbI_3_–CsPbCl_3_ as intermediates of the halide exchange reaction in our experiments. After complete halide exchange, the mismatch at the perovskite–chalcohalide interface is ≈8.5% (Figure S9g,h,i, Supporting Information). Again, this value, obtained for the final heterostructures, includes compensation due to the presence of a Pb_4_S_3_(Cl_1−x_I_x_)_2_ layer in the chalcohalide region close to the interface. Experimentally, the interfacial atomic distances parallel to the perovskite–chalcohalide interface are measured to be 5.69 ± 0.08 Å for the initial heterostructures (Figure S9c, Supporting Information, black box) and 5.92 ± 0.18 Å for the fully exchanged ones (Figure S9i, Supporting Information, black box). These values, extracted from the spacing of the single atomic row at the interface, indicate that the perovskite lattice is compressed, whereas the chalcohalide lattice is expanded in order to maintain the epitaxial relation.

To shed light on the formation and stability of these intermediate multidomain structures, we performed a series of DFT simulations. Here, we mimic the experimental conditions by including PbI_2_ in the reaction equilibrium with the Cl‐based heterostructure, and we assume for simplicity that the displaced chlorine is released as PbCl_2_. Because of this, all Cl→I anion exchange reactions were described by the following exchange mechanism:

(2) $$HS\left(x\cdot Cl\right)+xPbI_{2}\to HS\left(x\cdot I\right)+xPbCl_{2}$$

where *HS*(*x* · *Cl*) and *HS*(*x* · *I*) are the NC heterostructure models before and after the exchange reaction, and *x* is the number of exchanged halides. The associated reaction energies were computed as:

(3) $$\Delta E_{exchange}=\left[\left(E_{HS\left(x\cdot I\right)}+E_{PbCl_{2}}\right)-\left(E_{HS\left(x\cdot Cl\right)}+E_{PbI_{2}}\right)\right]\;\;/x$$

that, they are normalized to the number of exchanged halide ions. We additionally computed the lattice strain on the perovskite subdomain from the Pb–Pb distances, which are directly related to the lattice vectors of the cubic perovskite structure. The strain was evaluated by comparing the Pb–Pb distances in the heterostructures to those in the corresponding standalone perovskite NC, according to the expression:

(4) $$\mathrm{strain}\;\;\left(\%\right)=\frac{d_{\mathrm{Pb}-\mathrm{Pb}}^{\mathrm{HS}}-d_{\mathrm{Pb}-\mathrm{Pb}}^{\mathrm{perovskite}}}{d_{\mathrm{Pb}-\mathrm{Pb}}^{\mathrm{perovskite}}}\cdot 100$$

**Figure 4:**
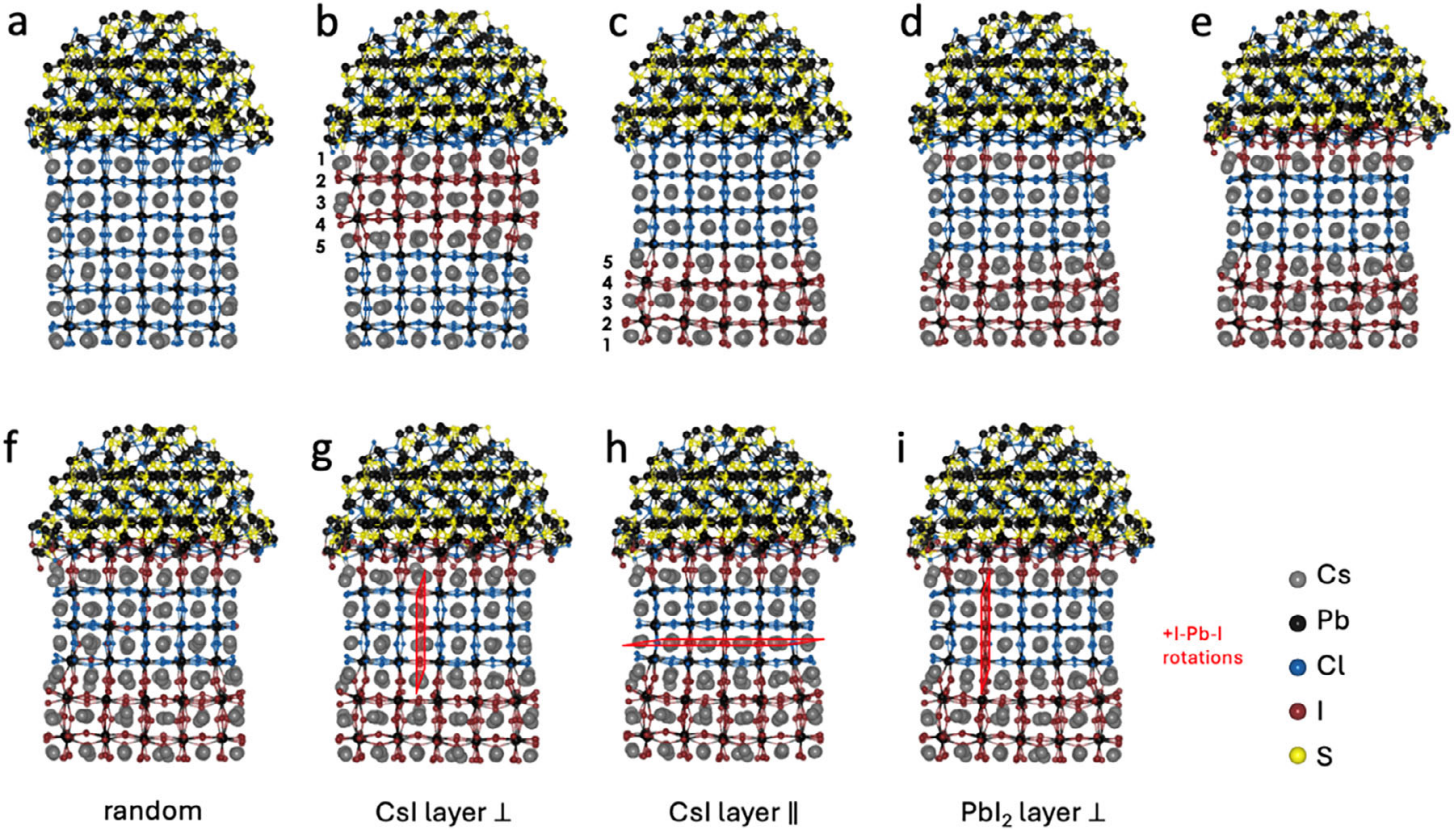
Ball and stick representation of heterostructure models optimized at the DFT/PBE level of theory (Cs^+^ = gray, Pb^2+^ = black, Cl^−^ = blue, I^−^ = red, S^2−^ = yellow). a) pristine CsPbCl_3_–Pb_4_S_3_Cl_2_; b,c) anion exchanged samples in five perovskite layers, respectively starting from the CsPbCl_3_–Pb_4_S_3_Cl_2_ interface or from the bottom of the perovskite domain, the latter being slightly favored energetically. For model (c), further anion exchange reactions were modelled at the interface, including d) full exchange of a CsCl monolayer in the perovskite domain, and e) additional partial exchange of ≈60% of Cl with I in two layers of the chalcohalide domain, reducing the interfacial strain. f–i) Model (d) after exchanging ≈20% of the Cl^−^ ions with I^−^ ions in the CsPbCl_3_ domain. Four possible distributions of the I^−^ ions were probed: (f) randomly in the CsPbCl_3_ domain; ordered (g) in a CsI layer perpendicular to the interface, (h) in a CsI layer parallel to the interface and (i) in a PbI_2_ layer perpendicular to the interface. The ordered configurations are always preferred over the random ones.

In this framework, the interfacial strain is defined as the average absolute value of the strain on the Pb–Pb distances within the interfacial perovskite layer. We started from the CsPbCl_3_–Pb_4_S_3_Cl_2_ NC heterostructure model published previously by us (**Figure** **4**a)^[^ ^14^ ^]^ and gradually replaced Cl^−^ ions with I^−^ ions in five layers of the perovskite domain, either starting from the CsPbCl_3_–Pb_4_S_3_Cl_2_ interface (Figure 4b) or from the bottom of the perovskite domain (Figure 4c). A glance at energies associated with the Cl→I exchange in each layer, reported in Table S1 (Supporting Information), highlights that the partial substitution of Cl^−^ with I^−^ has a slight preference for the bottom of the perovskite domain, favoring the formation of a segmented CsPbI_3_–CsPbCl_3_–Pb_4_S_3_Cl_2_ NC heterostructure, as observed in the experiments. This pathway is not only energetically favored but also maintains a low interfacial strain on the perovskite (1.57% for Figure 4c), comparable to the unexchanged heterostructure (1.66%), whereas the alternative pathway starting from the interface results in a higher strain (2.04% for Figure 4b).

We then investigated the structural and energetic changes associated with the partial substitution of Cl^−^ with I^−^ at the interface between the perovskite and chalcohalide domains. We started from the CsPbI_3_–CsPbCl_3_–Pb_4_S_3_Cl_2_ NC heterostructure model of Figure 4c and fully replaced Cl^−^ with I^−^ in the interfacial layer of the perovskite domain (single monolayer). This is represented in Figure 4d and essentially reproduces the interfacial CsI layer observed in the CNN image of Figure 3b,c. We then gradually replaced Cl^−^ ions with I^−^ ions in the first two layers of the chalcohalide domain, achieving in these layers I/Cl ratios of 20%, 40%, 60%, 80%, and 100%, respectively (Figure 4e). As shown by the energy values reported in Table S2 (Supporting Information), the initial introduction of I^−^ ions into the interfacial chalcohalide layers (from 0% to 20%) is the most energetically favored process, an effect that we ascribe to a substantial relaxation of the interfacial strain. Thereafter, the enthalpy change remains essentially constant as the I/Cl ratio increases. We note that entropic contributions, which are not included in our calculations, may play a critical role in stabilizing intermediate I/Cl ratios at ≈40–60% (Figure 4e), as the number of possible configurations for halide ion distribution is maximized in this range. This mixed distribution is in line with the intensities of the halide columns observed in the CNN images.

Finally, we simulated the formation of an I:CsPbCl_3_ alloy by substituting ≈20% of the Cl^−^ ions with I^−^ ions in the CsPbCl_3_ domain. Guided by the electron microscopy analysis (Figure 2), we decided to probe four possible distributions of the I^−^ ions: randomly in the CsPbCl_3_ domain (Figure 4f); ordered in a CsI layer perpendicular to the interface (Figure 4g), in a CsI layer parallel to the interface (Figure 4h) and in a PbI_2_ layer perpendicular to the interface (Figure 4i). As reported in Table S3 (Supporting Information), the formation of ordered I:CsPbCl_3_ configurations is always preferred, with a stabilization energy of ≈5–15 kcal mol^−1^ over the random configuration, which corresponds to a stabilization of 0.33–1.00 kcal mol^−1^ per atom being exchanged. The total stabilization energy is expected to be amplified in the NC heterostructures of experimental size, where a larger number of halide ions are exchanged. A closer look at the relaxed structure of Figure 4g,i reveals how ordered arrangements of I^−^ ions can result in the expansion of the CsPbCl_3_ lattice parallel to the interface (due to the elongation of the X─Pb─X bonds), ultimately decreasing the strain at the CsPbCl_3_–CsPbI_3_ interface. These results help us rationalize the ordered arrangement of I^−^ ions in perpendicular layers within the CsPbCl_3_ domain observed in the electron microscopy images (green arrow in Figure 2b).

**Figure 5:**
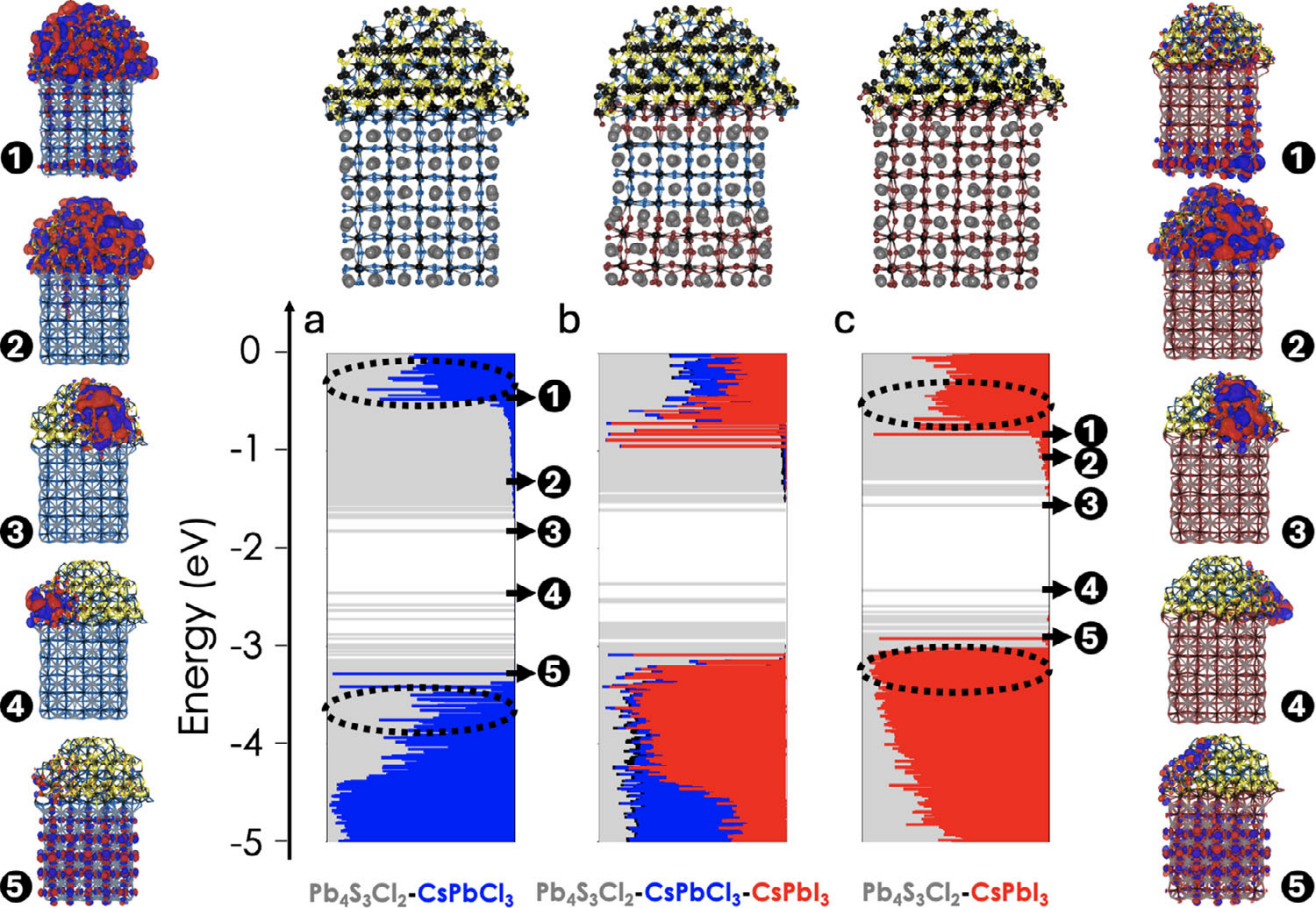
The electronic structure of a) the CsPbCl_3_–Pb_4_S_3_Cl_2_, b) the CsPbI_3_–CsPbCl_3_–Pb_4_S_3_Cl_2,_ and c) the CsPbI_3_–Pb_4_S_3_Cl_2_ heterostructures computed at the DFT/PBE level of theory. For each molecular orbital (MO), the length of the different line sections represents the fractional contribution from the chalcohalide (gray), CsPbCl_3_ (blue), and CsPbI_3_ (red) domains, respectively. Isosurfaces of the most relevant molecular orbitals are additionally reported for the parent and fully exchanged heterostructures with a counter value of 0.02 e Bohr^−3^, highlighting positive and negative parts in red and blue, respectively. MO1 and MO5 highlight delocalized perovskite contributions in both CB and VB; MO2 illustrates delocalized chalcohalide contributions in the CB; MO3 and MO4 represent the CB and VB edges of the heterostructures, defined by trap states localized at the surface of the chalcohalide domain.

We then computed the electronic structure of models of the starting CsPbCl_3_–Pb_4_S_3_Cl_2_ (Figure 4a),^[^ ^14^ ^]^ the partially exchanged CsPbI_3_–CsPbCl_3_–Pb_4_S_3_Cl_2_ (Figure 4e) and the fully exchanged CsPbI_3_–Pb_4_S_3_Cl_2_ heterostructures. The results are reported in **Figure** **5**a–c. In all the cases, the valence band (VB) and conduction band (CB) edges of the heterostructure are defined by surface trap states on the chalcohalide domain (gray lines in the density of states, molecular orbital plots (3) and (4)), a result that is consistent with our previous work on the CsPbCl_3_–Pb_4_S_3_Cl_2_ heterostructures.^[^ ^14^ ^]^ For CsPbCl_3_–Pb_4_S_3_Cl_2_, the energetic alignment between the bands of Cl‐based perovskite domain and the bands of the chalcohalide domain gives rise to a mixed perovskite–chalcohalide configuration in both CB and VB (dashed ovals in Figure 5a). The driving force provided by the energy level alignment of these traps, together with the orbital overlap between the two domains, is expected to lead to efficient transfer of photoexcited electrons and holes to chalcohalide trap states. This enables non‐radiative recombination pathways, explaining the lack of emission from the starting CsPbCl_3_–Pb_4_S_3_Cl_2_ heterostructure. In contrast, for the fully exchanged CsPbl_3_–Pb_4_S_3_Cl_2_ heterostructure, the band misalignment results in VB states localized on the CsPbl_3_ domain, while CB states remain mixed (dashed ovals in Figure 5c). Here, photo‐excited holes are more likely to radiatively recombine with the electrons in the perovskite domain, accounting for the observed emission from the partially exchanged CsPbI_3_–CsPbCl_3_–Pb_4_S_3_Cl_2_ and fully exchanged CsPbI_3_–Pb_4_S_3_Cl_2_ heterostructures. Additionally, in the partially exchanged case, the presence of the CsPbCl_3_ domain prevents (both physically and electronically) the transfer of photo‐excited electrons and holes from the CsPbl_3_ to the chalcohalide states, further facilitating the radiative recombination of the charge carriers in the I‐based perovskite domain. We note that these considerations are based on ground‐state DFT and should therefore be regarded as qualitative trends; more rigorous insight into excited‐state recombination dynamics would require TDDFT or related approaches, which remain computationally prohibitive for nanocrystal models of this size.

**Figure 6:**
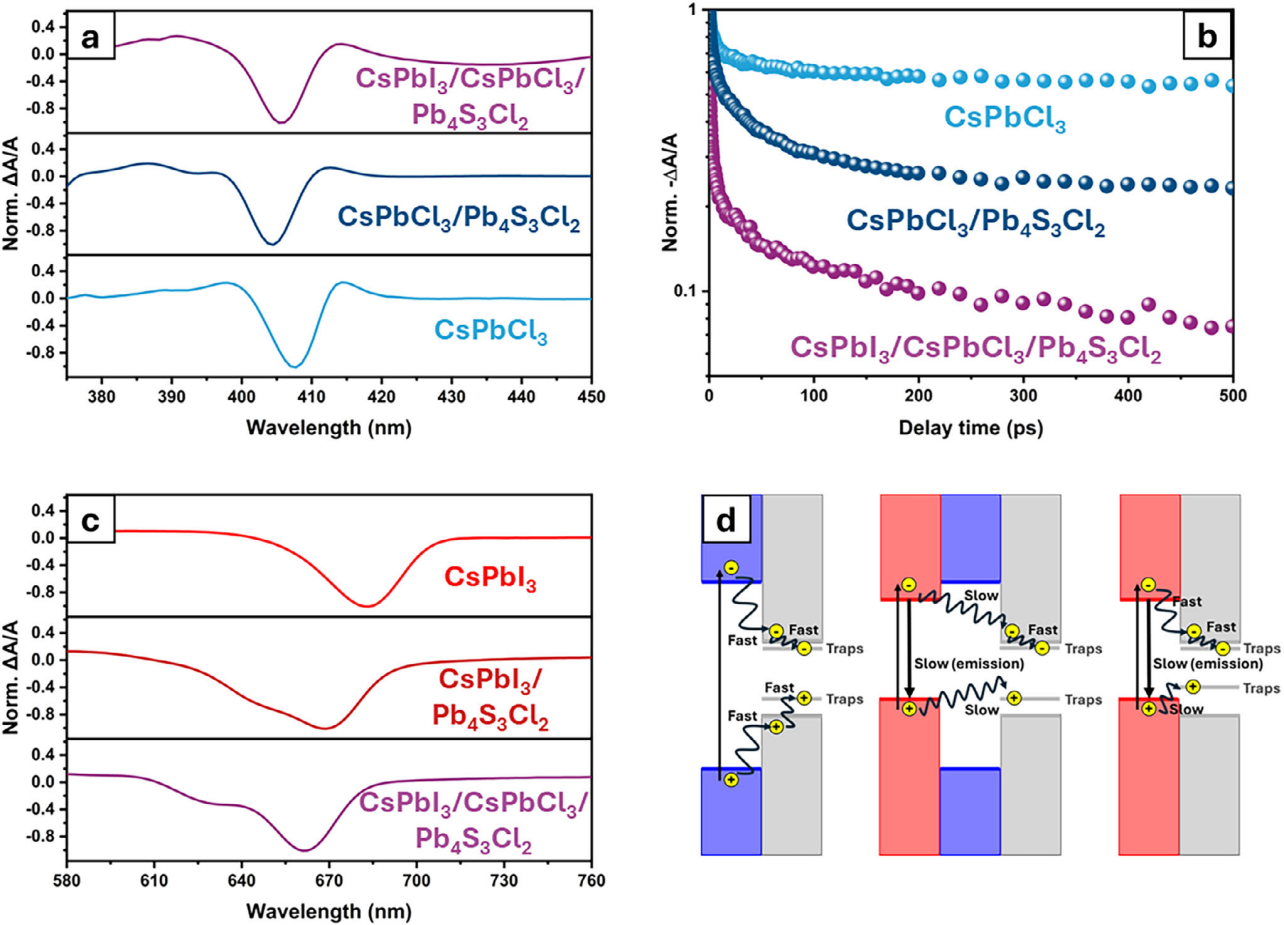
a) Transient absorption spectra at 1 ps delay for CsPbCl_3_, CsPbCl_3_–Pb_4_S_3_Cl_2_, and CsPbI_3_–CsPbCl_3_–Pb_4_S_3_Cl_2_ heterostructures excited at 370 nm. b) Decay profiles of CsPbCl_3_ exciton GSB in each sample. c) Transient absorption spectra at 1 ps delay for the CsPbl_3_, CsPbl_3_–Pb_4_S_3_Cl_2_, and CsPbI_3_–CsPbCl_3_–Pb_4_S_3_Cl_2_ heterostructures. d) Schematic band‐diagram of the heterostructures (CsPbCl_3_ in light blue; Pb_4_S_3_Cl_2_ in gray; CsPbl_3_ in light red) illustrating the possible charge transfers under 370 and 500 nm excitation.

We carried out transient absorption (TA) spectroscopy on the starting, intermediate and fully exchanged heterostructures, as well as on free‐standing CsPbCl_3_ and CsPbI_3_ NCs for comparison. The study focused on ground‐state bleaching (GSB) of CsPbCl_3_ and CsPbI_3_ excitons as key indicators of carrier behavior. Two different wavelengths of the laser pump were systematically employed: 370 nm, which is above the bandgap of both CsPbCl_3_ and CsPbI_3_ domains, and 500 nm, which selectively excites only that of CsPbI_3_. **Figure** **6**a presents the TA spectra at a 1 ps delay for CsPbCl_3_ NCs, initial CsPbCl_3_–Pb_4_S_3_Cl_2_ and intermediate CsPbI_3_–CsPbCl_3_–Pb_4_S_3_Cl_2_ heterostructures. In agreement with the steady‐state absorption measurements (Figure 1a; Figure S10, Supporting Information), the GSB signal of the CsPbCl_3_ exciton is observed ≈405 nm in the heterostructures and 408 nm for the CsPbCl_3_ NCs. Furthermore, positive features on both the higher‐ and lower‐energy sides of the exciton GSB are attributed to photoinduced absorption (PA) processes and biexciton signals, respectively.^[^ ^21^, ^22^, ^23^, ^24^ ^]^ Figure 6b shows the decay profiles of exciton GSB for the CsPbCl_3_ domain in each sample. The reference free‐standing NCs (cyan trace) exhibit a significantly slower decay rate, with long exciton recombination times (nanosecond timescale). In contrast, the CsPbCl_3_ exciton GSB in the starting CsPbCl_3_–Pb_4_S_3_Cl_2_ heterostructure decays more rapidly (blue trace), an effect that can be attributed to the formation of a type‐I alignment between these domains.^[^ ^14^ ^]^ A faster GSB decay compared to the free‐standing case would indeed result from the transfer of carriers from the CsPbCl_3_ domain to the Pb_4_S_3_Cl_2_ one upon photoexcitation. The decay dynamics of the CsPbCl_3_ exciton GSB in the pristine CsPbCl_3_–Pb_4_S_3_Cl_2_ heterostructures suggest a multiexponential behavior, where the fast components are related to charge transfer to the Pb_4_S_3_Cl_2_ domain and the slow component can be assigned to exciton recombination in the CsPbCl_3_ region. Upon partial exchange and the formation of a CsPbI_3_ domain in the heterostructure, the CsPbCl_3_ exciton GSB decays at an even faster rate (purple line), suggesting that most of the excited charge carriers in the CsPbCl_3_ domain are quickly transferred across the CsPbCl_3_–CsPbI_3_ interface.

The carrier dynamics can also be analyzed from the perspective of the CsPbI_3_ domain. Within this context, Figure 6c presents the TA spectra at a 1 ps delay for the CsPbl_3_ NCs, CsPbl_3_–Pb_4_S_3_Cl_2_, and CsPbI_3_–CsPbCl_3_–Pb_4_S_3_Cl_2_ heterostructures. The GSB of CsPbl_3_ excitons was observed at a shorter wavelength for CsPbI_3_–Pb_4_S_3_Cl_2_ (≈670 nm) and CsPbI_3_–CsPbCl_3_–Pb_4_S_3_Cl_2_ (≈650 nm) compared to the isolated CsPbI_3_ NCs (680 nm), in line with the steady‐state PL and absorption data reported in Figure 1a and Figure S10 (Supporting Information). This suggests possible differences in NC size and/or the presence of Cl^−^ ions in the CsPbl_3_ domain. Concurrently, bleach signals at ≈620 nm were retrieved in the partially exchanged sample, which in turn could reflect the presence of alloying or populations of exchanged nano‐heterostructures at different stages of exchange within the sample. The observation of GSB of CsPbI_3_ (Figure 6c) and CsPbCl_3_ (Figure 6a) excitons, together with the fast decay of CsPbCl_3_ exciton GSB in the partially exchanged heterostructures, is another indication of the presence of a CsPbCl_3_–CsPbI_3_ interface, which further supports the electron microscopy data. In addition, the partially exchanged heterostructures exhibit a slower rise time of the CsPbI_3_ exciton GSB compared to the fully exchanged heterostructure under 370 nm pump illumination (Figure S11a, Supporting Information). This is in line with a charge transfer from the CsPbCl_3_ domain to the CsPbI_3_ domain.^[^ ^25^ ^]^ Conversely, when the pump excitation is below the CsPbCl_3_ bandgap (i.e. 500 nm), and only the CsPbI_3_ domain can be excited, the rise profile of the CsPbI_3_ exciton GSB is similar in both partially and fully exchanged heterostructures (Figure S11b, Supporting Information). The decay of CsPbI_3_ GSB (Figure S12a,b, Supporting Information) is faster in the case of the fully exchanged sample compared to both the partially exchanged heterostructure and free‐standing CsPbI_3_ NCs. Additional processes at the newly formed CsPbI_3_–Pb_4_S_3_Cl_2_ interface are indeed expected from the band structure calculations in a fully exchanged heterostructure, where carrier trapping due to defects could result in a faster decay of CsPbI_3_ exciton GSB (Figures 5c and 6d). In the case of a partially exchanged sample, the presence of the CsPbCl_3_ domain hinders any such process, and this is also supported by the calculations presented above. Figure 6d provides a schematic energy diagram illustrating the system: the wide‐bandgap CsPbCl_3_ domain (light blue) is centrally located, and, on either side, there are narrower bandgap Pb_4_S_3_Cl_2_ (gray) and CsPbI_3_ (light red) domains. Upon photoexcitation, charge transfer occurs preferentially and more rapidly to the CsPbI_3_ domain, highlighting its efficient charge transfer properties. The CsPbCl_3_ domain also acts as an energy barrier between the Pb_4_S_3_Cl_2_ and CsPbI_3_ domains, facilitating the radiative recombination in the CsPbI_3_ region.

Finally, time‐dependent stability tests were performed on the fully exchanged CsPbI_3_–Pb_4_S_3_Cl_2_ heterostructures. The results are reported in Figure S13 (Supporting Information). The heterodimer morphology was preserved for the first eight days, with no evidence of secondary phases in either optical absorption or photoluminescence spectra. At longer times, however, heterodimers were no longer observed, and mostly isolated particles were present. This reduced stability is consistent with the large lattice mismatch between CsPbI_3_ and Pb_4_S_3_Cl_2_, which limits the long‐term preservation of a stable epitaxial interface.

## 3 Conclusion

This study demonstrates that CsPbCl_3_–Pb_4_S_3_Cl_2_ NC heterostructures enable controlled Cl→I halide exchange, unlike the rapid, limited alloying in free‐standing CsPbCl_3_ NCs. The epitaxial perovskite–chalcohalide interface directs a gradual exchange, with iodide ions substituting chloride ions in the perovskite domain farthest from the interface, forming segmented CsPbI_3_–CsPbCl_3_–Pb_4_S_3_Cl_2_ heterostructures with a unique CsPbI_3_–CsPbCl_3_ epitaxial interface. DFT calculations confirm that this configuration minimizes the total energy of the system, which it is further stabilized by iodine incorporation at the perovskite–chalcohalide interface. As the exchange progresses, the CsPbCl_3_ domain shrinks to a few unit cells in projection and exhibits iodine alloying, with I^−^ ions preferentially aligned perpendicular to the CsPbCl_3_–CsPbI_3_ interfaces. These configurations also correspond to energetically favorable states, as supported by computational modeling. Optical analyses reveal type‐I band alignments in intermediate heterostructures (both at the CsPbCl_3_–CsPbI_3_ and CsPbCl_3_–Pb_4_S_3_Cl_2_ interfaces). Especially for the CsPbCl_3_–CsPbI_3_ interface, this band alignment enables efficient carrier transfer to the CsPbI_3_ domain, resulting in red emission.

In conclusion, the CsPbCl_3_–Pb_4_S_3_Cl_2_ epitaxial heterostructures strongly influence the Cl→I exchange mechanism, compared to free‐standing CsPbCl_3_ NCs, leading to novel segmented heterostructures sustaining a CsPbI_3_–CsPbCl_3_ interface, despite significant lattice mismatch between these two halide perovskite lattices. This behavior positions such heterostructures as a promising platform to induce controlled phase segregation and even create heterostructures with substantial lattice mismatch that are otherwise impossible to achieve via direct synthesis or by post‐synthesis anion exchange in free‐standing NCs. Such tunability in phase and band alignment can be further exploited in light‐emitting applications, while engineering a type‐II alignment promoting charge separation could specifically benefit photocatalysis or photovoltaics, offering a versatile platform for advanced optoelectronic and photocatalytic materials.

## 4 Experimental Section

### Materials

Cesium carbonate (Cs_2_CO_3_, 99,9%), lead chloride (PbCl_2_, >98%), lead iodide (PbI_2_, >98%), lead acetate trihydrate (Pb(CH_3_COO)_2_·3H_2_O, 99.99%), dodecanethiol (DDT, 99.9%), elemental sulfur (S, >99%), 1‐octadecene (ODE, C_18_H_36_, 90%), oleic acid (OA, C_18_H_34_O_2_, 90%), oleylamine (OLAm, C_18_H_37_N, 70%), anhydrous toluene (C_7_H_8_, >99.8%), ethyl acetate (C_4_H_8_O_2_), fumed silica powder (SiO_2_), and hexane (99.8%) were purchased from Merck. All chemicals were used without further purification.

### PbCl_2_ Stock Solution

PbCl_2_ (2 mmol), 30 mL of octadecene, 5 mL of oleylamine, and 5 mL of oleic acid were loaded in a 100 mL 3‐neck flask. The mixture was first degassed for 30 min at room temperature and then for 30 min at 110 °C under stirring. Finally, the solution was heated up to 150 °C under N_2_ until the salt was completely dissolved. The resulting solution was transferred into an N_2_‐filled glass vial and was stored inside the glovebox for further use.

### Cs‐OL Stock Solution

Cs_2_CO_3_ (1 mmol), 2.5 mL of oleic acid, and 8.75 mL of octadecene were loaded in a 25 mL 3‐neck flask. The mixture was first degassed for 30 min at room temperature and then for 1 hat 110 °C under stirring. The resulting solution was transferred into an N_2_‐filled glass vial and was stored inside the glovebox for further use.

### Pb‐OL Stock Solution

1 mmol of lead acetate trihydrate, 650 µL of oleic acid, and 9.35 mL of octadecene were loaded in a 50 mL 3‐neck flask. The mixture was first degassed for 30 min at room temperature and then for 1 h at 110 °C under stirring. The resulting solution was transferred into an N_2_‐filled glass vial and was stored inside the glovebox for further use.

### S‐ODE Precursor Solution

0.5 mmol of sulfur powder and 5 mL ODE (previously degassed for 1 h at 110 °C) were loaded in a 7 mL vial inside the glovebox. The resulting mixture was sonicated until the sulfur powder was completely dissolved.

### PbI_2_ Stock Solution

1 mmol of PbBr_2_, 2.5 mL of oleic acid, 2.5 mL of oleylamine, and 15 mL of octadecene were loaded in a 40 mL vial. The mixture was degassed for 30 min at room temperature and then for 30 min at 110 °C. Then, the solution was heated up to 150 °C under N_2_ until the salt was completely dissolved. The resulting solution was transferred into an N_2_‐filled glass vial and was stored inside a glovebox for further use.

### Oleylammonium Iodide Precursor Solution

I_2_ powder (6 mmol), oleylamine (0.027 mol), and ODE (21 mL) in a 40 mL glass vial. The vial was placed on the hot plate and degassed under vacuum for 30 min at 110 °C. The resulting solution was cooled down at room temperature and stored under nitrogen.

### CsPbCl_3_ Clusters Synthesis

CsPbCl_3_ clusters were synthesized following a previously reported method with slight modifications.^[^ ^14^ ^]^ Briefly, 4 mL of the PbCl_2_ stock solution was transferred into a 20 mL vial. The solution was heated up to 50 °C and then 0.25 mL of Cs‐OL stock solution was injected into the PbCl_2_ stock solution. The resulting mixture was kept under stirring at 50 °C for 30 min. The resulting solution was purified by centrifugation (8000 rpm, 5 min), and the supernatant was discarded. Finally, the precipitate was redispersed in a solution containing 0.9 mL of previously degassed octadecene.

### CsPbCl_3_‐Pb_4_S_3_Cl_2_ NC Heterostructures Synthesis

In a typical synthesis, 4.0 mL of previously degassed octadecene was loaded into a 20 mL N_2_‐filled vial. Then, the vial was heated up at 200 °C for 5 min. After this time, the following precursor solutions were injected into octadecene: 0.1 mL of Pb‐OL solution, 0.2 mL of DDT‐ODE solution (500 uL DDT and 4.5 mL of previously degassed octadecene), clusters solution (mentioned above), and 0.1 mL of sulfur source (S‐ODE). The reaction was kept under stirring for the corresponding reaction time of 5 min and finally quenched in an ice‐water bath. Purification steps included the addition of ethyl acetate (3 mL) into the crude solution and centrifugation (6000 rpm, 5 min). Afterward, the supernatant was discarded, and the precipitate was redispersed in 2 mL of toluene. To assess the concentration, the optical absorption spectrum of the NC solution was measured after diluting 25 µL of the NC solution to 2.5 mL toluene, resulting in an optical density of 0.14 at 370 nm.

### Synthesis of CsPbCl_3_ NCs

CsPbCI_3_ colloidal NCs, were synthesized by injecting the previously made CsPbCI_3_ clusters (dispersed in 0.9 mL ODE) in 4 mL pre‐degassed ODE at 160 °C. The reaction was quenched by an ice/water bath after 10 min. Then, 2.5 mL of anhydrous methyl acetate was added to the crude solution, and the solution was centrifuged for 5 min at 6000 rpm. Finally, the precipitate was redispersed in toluene

### Synthesis of CsPbI_3_ NCs

CsPbI_3_ colloidal NCs, were synthesized following the previously reported process.^[^ ^26^ ^]^ Cesium carbonate (16 mg), lead acetate trihydrate (76 mg), OA (0.2 ml) and octadecene (5 ml) were added in a 40 ml glass vial. The solution dried for 1 h at 100 °C. Then, the temperature was adjusted at 165 °C, and 2 ml of previously made oleylammonium iodide solution was swiftly injected under nitrogen. The reaction was quenched by an ice/water bath after 10 s. Then, 5 mL of anhydrous methyl acetate was added to the crude solution, and the solution was centrifuged for 5 min at 6000 rpm. Finally, the precipitate was redispersed in toluene.

### Cl→I Exchange Reactions

Halide exchange reactions were performed in a glovebox. In a typical reaction, 1 mL of the CsPbCl_3_–Pb_4_S_3_Cl_2_ solution was used for each reaction, and different amounts of PbI_2_ solution (ranging from 20 to 300 µL) were added under stirring at room temperature. When the reaction reached equilibrium, 600 µL of ethyl acetate was added to the solution. Finally, the NCs were collected by centrifugation at 6000 rpm for 5 min and redispersed in anhydrous toluene for further characterization.

### Optical characterization

Optical absorption spectra were collected on a Varian Cary 300 UV–vIS absorption spectrophotometer, while PL spectra were recorded by a Varian Cary Eclipse spectrophotometer using an excitation wavelength of 350 nm for the pristine sample and 500 nm for the exchanged samples. The NC solutions were diluted into toluene in quartz cuvettes (path length = 1 cm) to a maximum optical density below 1.0.

### X‐Ray Powder Diffraction

X‐ray powder diffraction measurements were performed on a PANanalytical Empyrean X‐ray diffractometer, equipped with a 1.8 kW Cu Kα ceramic anode and a PIXcel3D 2 × 2 area detector, operating at 45 kV and 40 mA. NC solutions were mixed with fumed silica and dried to minimize the preferential orientation. Finally, the powder was placed on a zero‐diffraction silicon substrate to perform the measurements.

### Scanning Transmission Electron Microscopy (STEM)

High‐resolution scanning TEM (HRSTEM) images were acquired on a probe‐corrected ThermoFisher Spectra 30–300 S/TEM operated at 300 kV, using a HAADF detector with a beam current of a few tens of pA to limit beam damage to the electron beam sensitive perovskite nanocrystals. The convergence angle was set to 25 mrad, corresponding to a sub‐angstrom electron beam. The calculated electron dose per frame was between 240 and 9500 e A^−2^ for HAADF‐STEM imaging. Compositional maps were acquired using Velox software, with a probe current of ≈150 pA and rapid rastered scanning. To improve the image quality of the elemental maps, a built‐in Gaussian blur filter with a sigma value of 1.0 pixels was applied. The energy‐dispersive X‐ray (EDX) spectroscopy signal was acquired on a Dual‐X setup comprising two detectors on either side of the sample, for a total acquisition solid angle of 1.76 Sr. The lattice parameters for HRSTEM images were mapped based on Pb atomic columns, positions of which were fitted using Gaussian functions.^[^ ^27^ ^]^

### Computational Methodology

To shed light on the structural and electronic properties of the NC heterostructures, we carried out atomistic simulations with a density functional theory (DFT) Hamiltonian. The Perdew–Burke–Ernzerhof (PBE) exchange‐correlation functional was employed.^[^ ^28^ ^]^ Scalar‐relativistic Goedecker–Teter–Hutter (GTH) pseudopotentials were used for all atoms, replacing the core electrons, while the valence electrons were described with a double‐ζ basis set plus polarization functions^[^ ^29^ ^]^ as implemented in the CP2K 2024.1 package.^[^ ^30^ ^]^ The auxiliary plane–wave cutoff was set to 400 Ry. All calculations were performed in vacuum within a non‐periodic simulation box of 52 × 68 × 52 Å. Structural relaxations were performed until the maximum residual force on atoms was below 4.5 × 10^−^⁴ Ha Bohr^−1^.

### 4D‐STEM Characterization

4D‐STEM imaging was performed with a probe‐corrected Thermo Fisher Titan Themis at an acceleration voltage of 200 kV and a semi‐convergence angle of the electron beam of 14 mrad. The NCs were deposited on a homemade graphene TEM‐grid.^[^ ^31^ ^]^ The 4D‐STEM datasets were acquired with a custom‐made Timepix3 detector2, which is an event‐driven hybrid pixelated direct electron detector, in event‐based format with 2048 × 2048 dimensions using a dwell time of 1 µs and a scanning step size of 0.1–0.2 Å. Final images were retrieved from 4D‐STEM datasets using a recently developed CNN approach.^[^ ^20^ ^]^ This method allows recovering the phase and the amplitude components of the exit electron wave based on a 3 × 3 kernel of adjacent convergent beam electron diffraction patterns. Real space was binned two times, and the detector was binned to 64 × 64 for the reconstruction. The data generation code is available open source under https://github.com/ThFriedrich/airpi. The electron doses were estimated by determining the number of detected events on the detector and by considering the cluster size. The cluster size correlates with the number of pixels which are excited by a single electron and was determined to be 2.3 for 200 kV.^[^ ^19^ ^]^ The octahedral tilt measurements were performed based on fitted Pb and X (halide) atomic columns with Gaussians by assessing the averaged angle between the lattice vectors and the vectors corresponding to the direction of PbX*6* octahedra, thus, reflecting the Pb‐Pb‐X angle.^[^ ^27^ ^]^ X‐X‐Cs angles analysis between two vectors was performed after fitting all atomic column positions with Gaussians and extracting the coordinates of required atomic columns (Cs and X).

### Transient Absorption Spectroscopy

A tunable Ytterbium‐based laser system (Pharos‐SP‐HP equipped with an optical parametric amplifier Orpheus from Light Conversion) was employed to perform the ultrafast transient absorption spectroscopy. The pulse duration of the pump was ≈160 fs, whereas the instant response function, determined by the cross‐correlation between pump and probe, was experimentally assessed to be ≈230 fs. An excitation pump power of ≈2 mW was used. All the TA spectra have been acquired through the Harpia‐TA (Light Conversion) system, where the supercontinuum white light is generated by focusing a portion of the second harmonic of the fundamental laser beam (at ≈515 nm) onto a sapphire crystal, and the delay between the pump and probe pulses has been tuned by changing the length of the probe optical path. The experiments were performed at 50 kHz, while the spot size of the pump/probe at the sample was measured to be ≈120 and 60 µm, respectively. Data from the ultrafast experiments were corrected for chirp dispersion using commercial software (CarpetView, Light Conversion).

## Conflict of Interest

The authors declare no conflict of interest.

## Supporting information



Supporting Information

## Data Availability

The data that support the findings of this study are available from the corresponding authors upon reasonable request.
