# Supplementary material for: Halide Perovskite–Chalcohalide Nanocrystal Heterostructures as a Platform for the Synthesis and Investigation of the CsPbCl3–CsPbI3 Epitaxial Interface
Source: Adv Mater. 2025 Nov 6;38(6):e12502. doi: 10.1002/adma.202512502 (PMC12848639; doi:10.1002/adma.202512502)
Supplement: Supplementary file 1 — Supporting Information [file ADMA-38-e12502-s001.docx]

Supporting Information

Halide Perovskite-Chalcohalide Nanocrystal Heterostructures as a Platform for the Synthesis and Investigation of the CsPbCl_3_-CsPbI_3_ Epitaxial Interface

Nikolaos Livakas, Irina Skvortsova, Juliette Zito, Yurii P. Ivanov, Aswin Asaithambi, Andrea Toma, Annick De Backer, Muhammad Imran, Sandra Van Aert, Giorgio Divitini, Ivan Infante*, Sara Bals* and Liberato Manna*


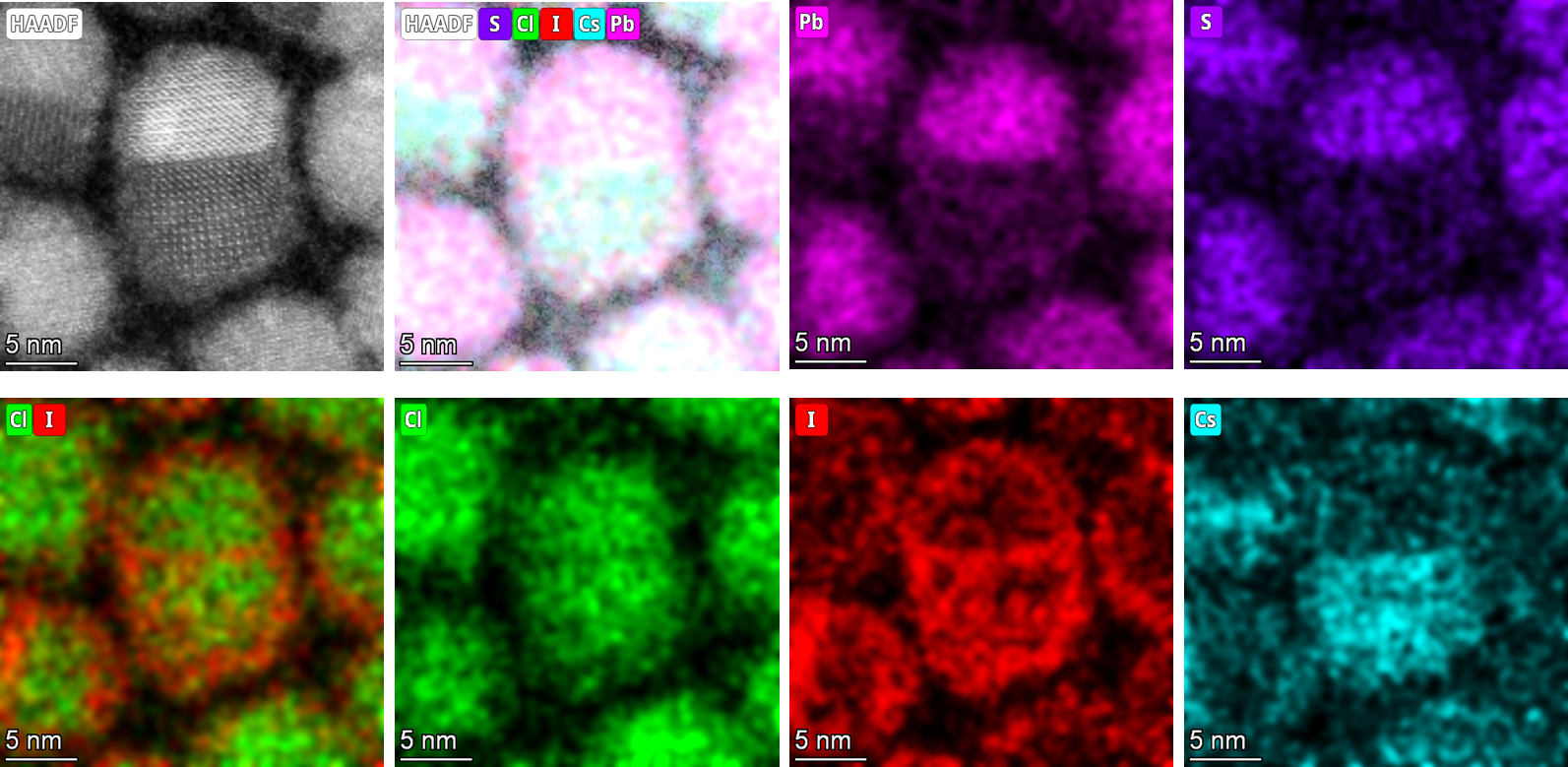


**Figure S1.** HAADF-STEM image of the partially exchanged segmented CsPbI_3_-CsPbCl_3_-Pb_4_S_3_Cl_2_ heterostructure with the corresponding STEM-EDX elemental maps.


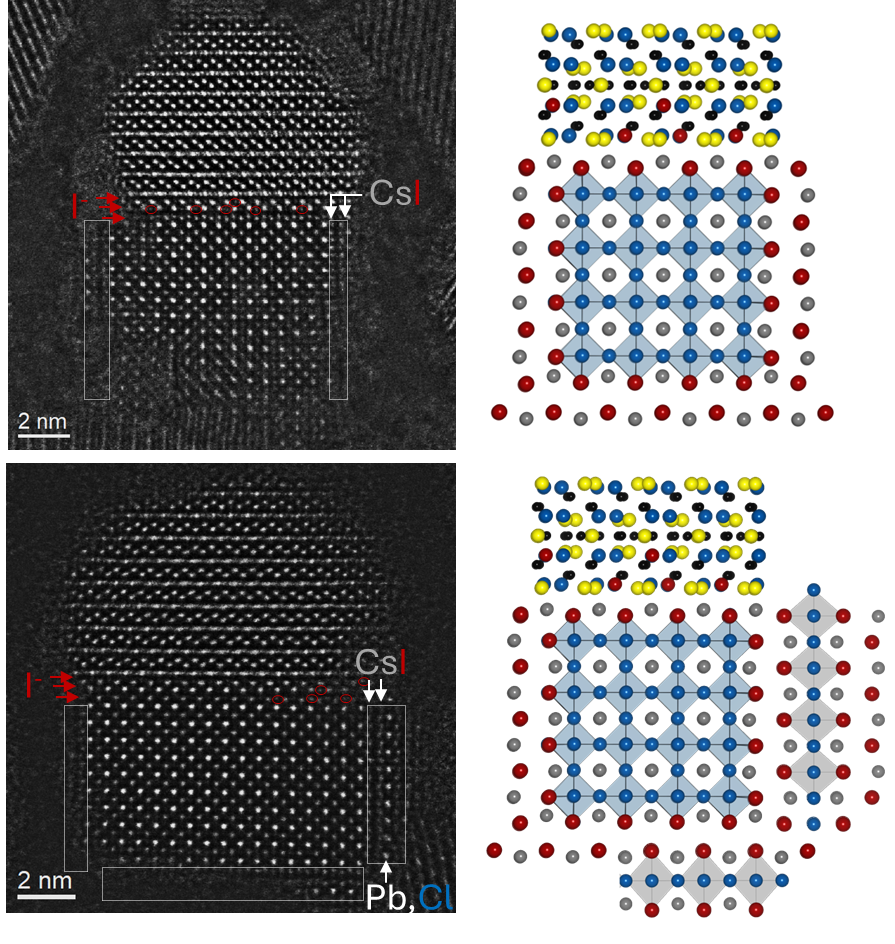


**Figure S2**. HAADF-STEM images of two nanocrystals captured at the early stages of the Cl→I exchange in CsPbCl₃–Pb₄S₃Cl₂ heterostructures. The early stage of the reaction may involve the formation of a CsI passivation layer on the CsPbCl₃ surface, accompanied by iodine incorporation at the perovskite–chalcohalide interface. The CsI surface layer, together with the underlying PbCl₂ layer of the CsPbCl₃ core, can be regarded as part of a Cs₂PbCl₂I₂ Ruddlesden–Popper (RP) phase, as in some cases nearly a complete RP unit cell is observed (bottom panel).


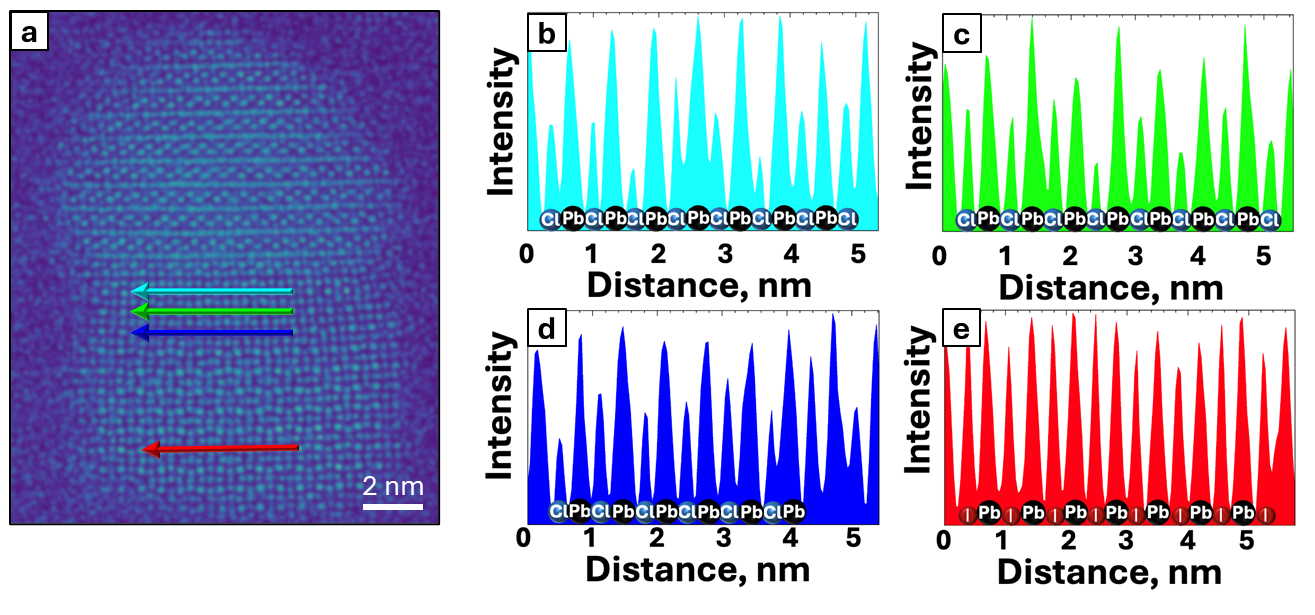


**Figure S3.** (a) Phase image obtained from CNN reconstruction of a 4D-STEM dataset of the partially exchanged CsPbI_3_-CsPbCl_3_-Pb_4_S_3_Cl_2_ heterostructures presented in Figure 3b. The arrows correspond to the intensity profiles presented in panels (b-e) highlighting the different perovskite subdomains (CsPbl_3_ at the bottom of the nanocrystal and CsPbCl_3_ close to the chalcohalide domain).


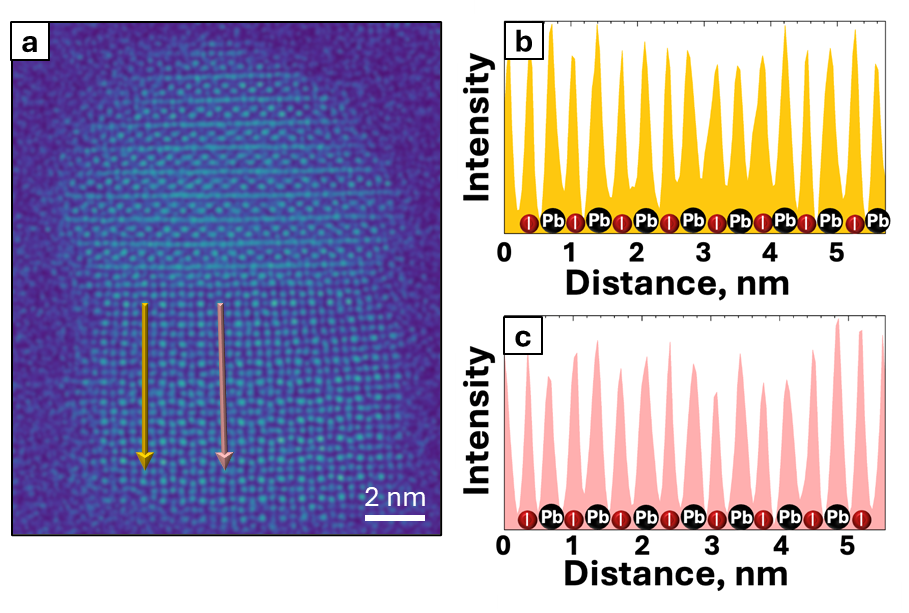


**Figure S4.** Phase image obtained from CNN reconstruction of a 4D-STEM dataset of the partially exchanged CsPbI_3_-CsPbCl_3_-Pb_4_S_3_Cl_2_ heterostructures presented in Figure 3b. The arrows correspond to the intensity profiles presented in panels (b and c) highlighting that the CsPbCl_3_ lattice is alloyed with iodine ions and those ions are concentrated in the axial direction.


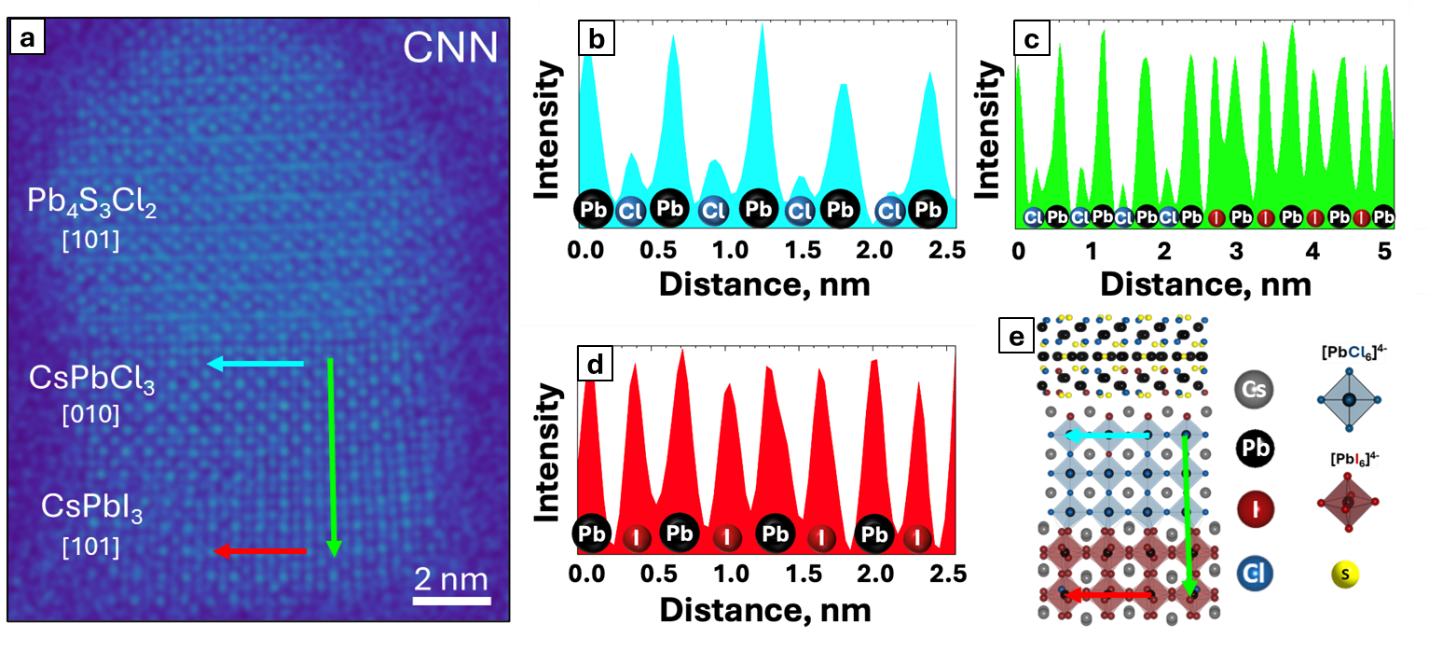
**Figure S5**. (a) Phase image obtained from CNN reconstruction of a 4D-STEM data set of the partially exchanged CsPbI_3_-CsPbCl_3_-Pb_4_S_3_Cl_2_ heterostructures with CsPbI_3_ aligned in [101] zone axis. Gaussian-filter was applied to the image. (b,c,d) Intensity profiles correspond to the arrows presented in panel (a). (e) Crystallographic model showcasing the multidomain structure presented in panel (a). In contrast to the intermediate case presented in Figure 3b, here the CsPbCl_3_ domain is bigger than few atomic rows, and there is no iodine alloying within its lattice.


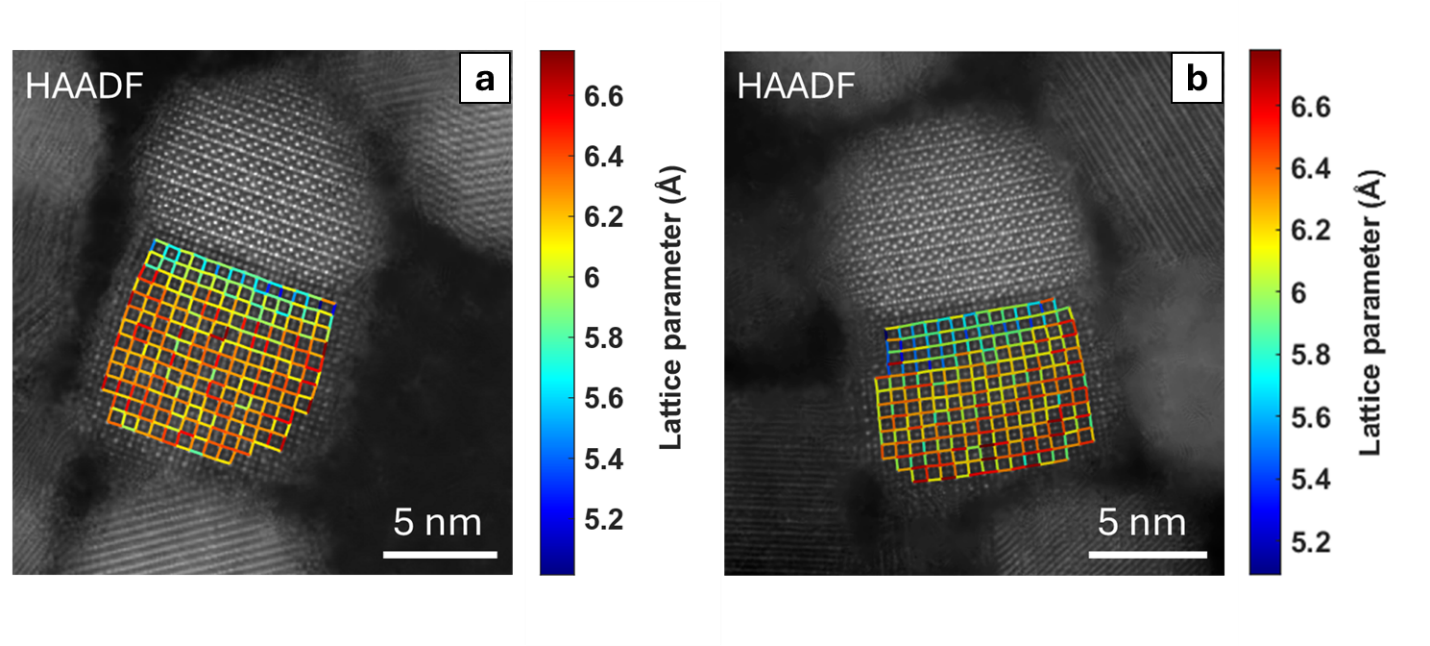


**Figure S6.** Lattice parameters quantified based on HAADF-STEM images of partially exchanged heterostructures, with (a) [010] oriented and (b) [101] oriented CsPbI_3_ domains.


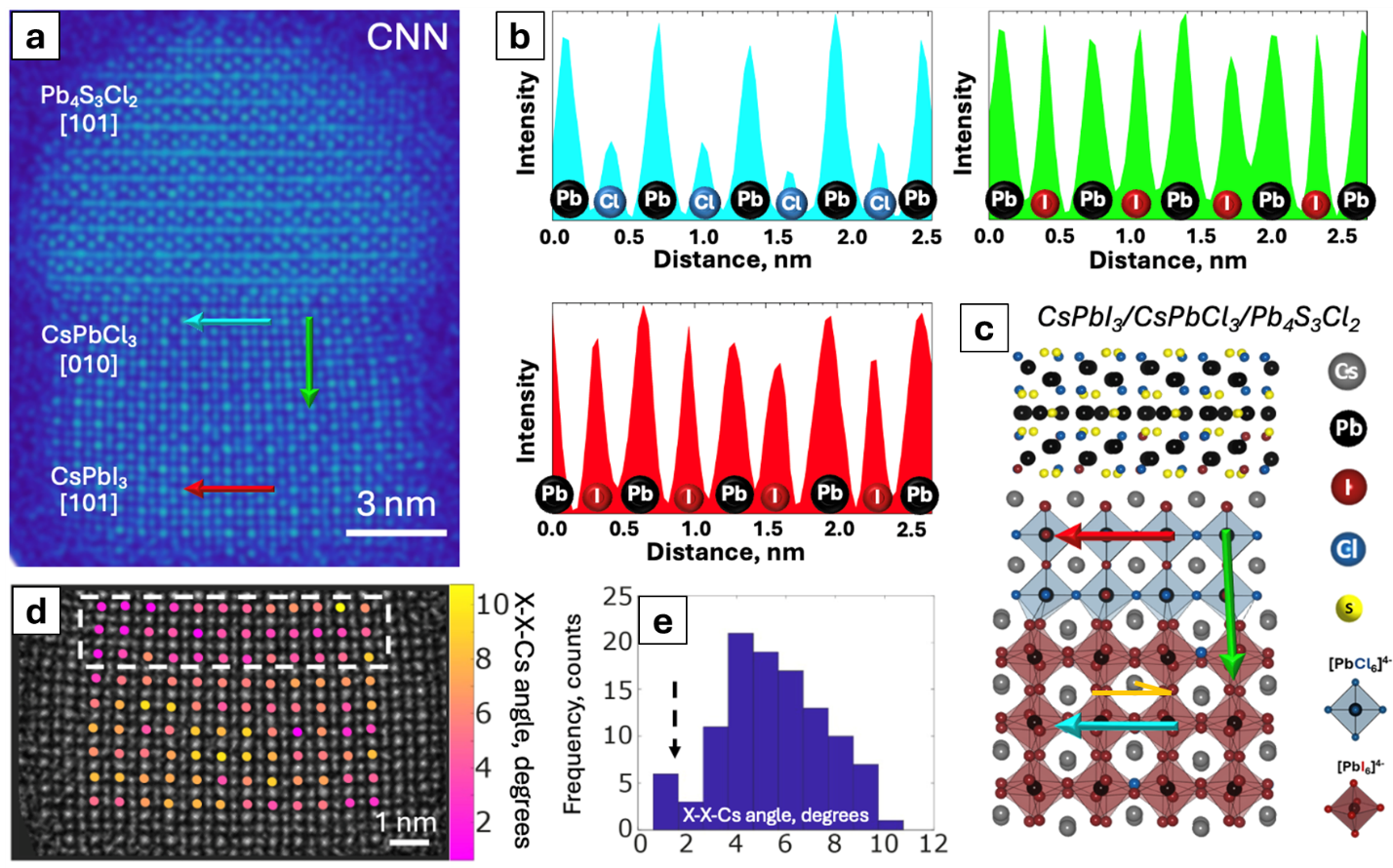


**Figure S7.** (a) Phase image obtained from CNN reconstruction of a 4D-STEM dataset of the partially exchanged CsPbI_3_-CsPbCl_3_-Pb_4_S_3_Cl_2_ heterostructures with CsPbI_3_ in [101] zone axis. Gaussian-filter was applied to the image. (b) Intensity profiles correspond to the arrows presented in panel (a). (c) Crystallographic model corresponding to the multidomain structure presented in panel (a). (d) Quantification of the X-X-Cs (X: halides) angles represented by a map of the angles throughout the perovskite domain for the partially exchanged sample. (e) Histogram of the measured X-X-Cs angles.

To quantify structural differences for the partially exchanged heterostructures with CsPbI_3_ in [101] orientation, we used X-X-Cs (where X corresponds to halides) angles as key indicators rather than measuring octahedral tilt (Pb-Pb-X angles) since the tilt cannot be not directly observed along this projection. The X-X-Cs values were compared for the intermediate CsPbCl_3_-CsPbI_3_-Pb_4_S_3_Cl_2_ (**Figure S7d,e**) and fully exchanged CsPbI_3_-Pb_4_S_3_Cl_2_ heterostructures (**Figure S8**). For the cubic CsPbCl_3_ lattice, which is expected to be present close to the interface with the chalcohalide domain, the angles are close to 0 degrees **(Figure S7e, black dashed arrow**). Again, when moving away from the interface the X-X-Cs angles increase up to 6-7 degrees. These values are in close agreement with those observed for the fully exchanged CsPbI_3_ (**Figure S8**). These observations therefore confirm the structural segmentation for the partially exchanged heterostructures.


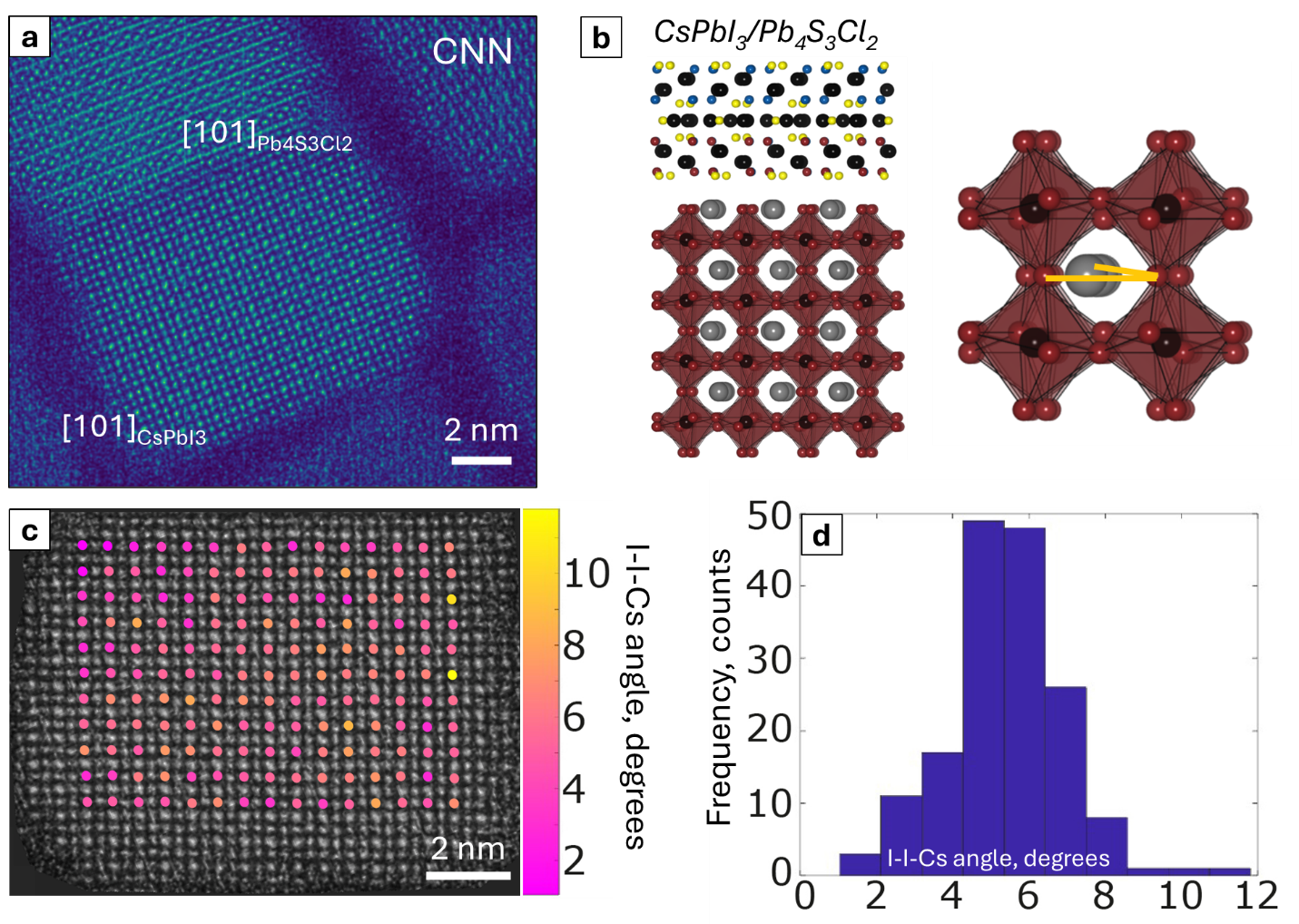


**Figure S8.** (a) Phase image obtained from CNN reconstruction of a 4D-STEM data set of the fully exchanged CsPbI_3_-Pb_4_S_3_Cl_2_ heterostructures with CsPbI_3_ in the [101] zone axis. Gaussian-filter was applied to the image. (b) Structural model corresponding to the image presented in panel (a). (c) Quantification of the I-I-Cs angles in the equatorial direction is represented by a map of the angles throughout the perovskite domain. (d) Histogram of the measured I-I-Cs angles.


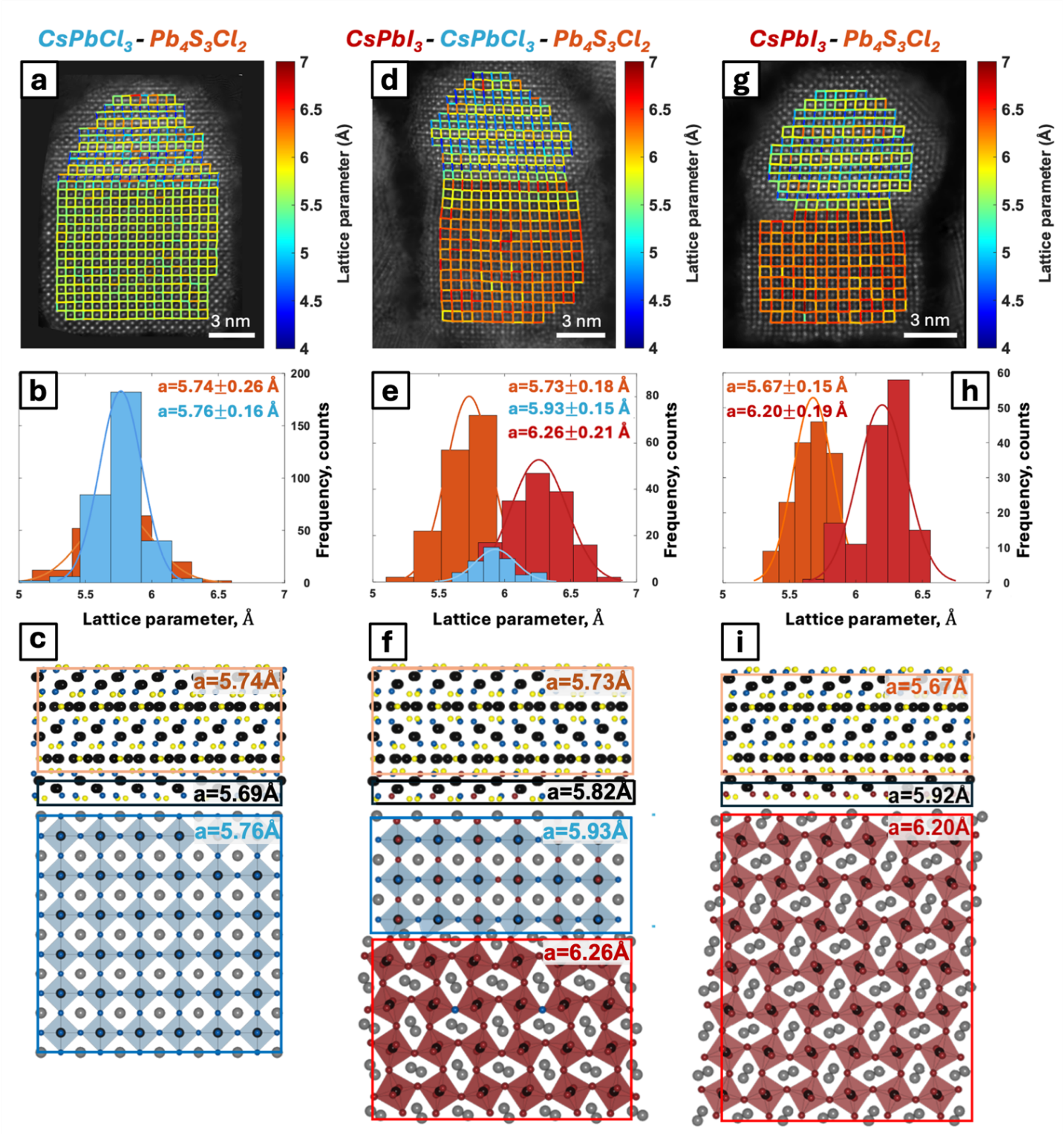


**Figure S9.** Lattice parameters analysis performed on HAADF-STEM images with corresponding distributions of the lattice parameters parallel to the perovskite-chalcohalide interface and structural models for (a,b,c) pristine, (d,e,f) partially and (g,h,i) fully exchanged heterostructures. Orange histograms and Gaussians belong to chalcohalide phase, blue histograms and curves correspond to CsPbCl_3_ part and red ones belong to CsPbI_3_ domain. For (e), the first three Pb-containing atomic rows in perovskite region (close to chalcohalide domain) are considered as CsPb(Cl_x_I_1-x_)_3_ and the rest is [010]-oriented CsPbI_3_. In (g) CsPbI_3_ domain is also oriented along the [010] direction. The average distances at the perovskite-chalcohalide interface are 5.69±0.08 Å for (a), 5.82±0.14 Å for (d) and 5.92±0.18 Å for (g), represented in black boxes in panels (c,f,i). This perovskite-chalcohalide interface corresponds to a monolayer with a Pb₄S₃Cl₂ composition for the pristine sample, and as Pb_4_S_3_(Cl_1-x_I_x_)_2_ composition for the partially and fully exchanged cases.

**Table S1.** Reaction energies associated to the Cl→I anion exchange in five layers of the perovskite domain, either starting from the CsPbCl_3_-Pb_4_S_3_Cl_2_ interface or from the bottom of the perovskite domain. All energies are normalized to the number of exchanged halide ions. In the last column, the positive sign indicates that the exchange is always more favored from the bottom.

| N layers | ΔE_exchange interface_ (kcal/mol) | ΔE_exchange_ _bottom_  (kcal/mol) | ΔE_exchange_ _interface_ –  ΔE_exchange_ _bottom_ (kcal/mol) |
| --- | --- | --- | --- |
| 1 | 4.32 | 0.53 | 3.78 |
| 2 | 3.79 | 3.39 | 0.40 |
| 3 | 4.11 | 3.58 | 0.53 |
| 4 | 3.80 | 3.54 | 0.26 |
| 5 | 3.95 | 3.71 | 0.25 |

**Table S2.** Reaction energies associated to the partial Cl→I anion exchange in two interfacial layers of the chalcohalide domain. All energies are normalized to the number of exchanged halide ions. In the third column, the positive sign indicates that the initial exchange (I/Cl ratio from 0% to 20%) is more favored than the subsequent exchanges. For each Cl→I exchange in the chalcohalide domain, the corresponding variation of the interfacial strain in the perovskite subdomain is also reported. The initial exchange (I/Cl ratio from 0% to 20%) corresponds to the largest release of interfacial strain.

| Exchange step (I/Cl ratio, %) | ΔE_exchange_ (kcal/mol) | ΔE_exchange_ - ΔE_exchange (0%→20%)_ (kcal/mol) | strain (%) |
| --- | --- | --- | --- |
| 0%→20% | 1.72 | 0.00 | 1.80%→1.58% |
| 20%→40% | 2.78 | 1.05 | 1.58%→1.44% |
| 40%→60% | 2.56 | 0.83 | 1.44%→1.45% |
| 60%→80% | 2.58 | 0.85 | 1.45%→1.40% |
| 80%→100% | 2.82 | 1.09 | 1.40%→1.51% |

**Table S3.** Reaction energies associated to the partial Cl→I anion exchange in the CsPbCl_3_ domain. Four possible distributions of the I ions are reported: random in the CsPbCl_3_ domain; ordered in a CsI layer perpendicular to the interface; ordered in a CsI layer parallel to the interface; ordered in a PbI_2_ layer perpendicular to the interface. In the last column, the negative sign indicates that the ordered configurations are always preferred over the random configuration.

| I distribution | ΔE_exchange_ (kcal/mol) | ΔE_exchange_ - ΔE_exchange (random)_ (kcal/mol) |
| --- | --- | --- |
| random | 87.13 | 0 |
| CsI layer ⊥ | 83.91 | -3.22 |
| CsI layer ∥ | 86.95 | -0.18 |
| PbI2 layer ⊥ | 70.38 | -16.75 |


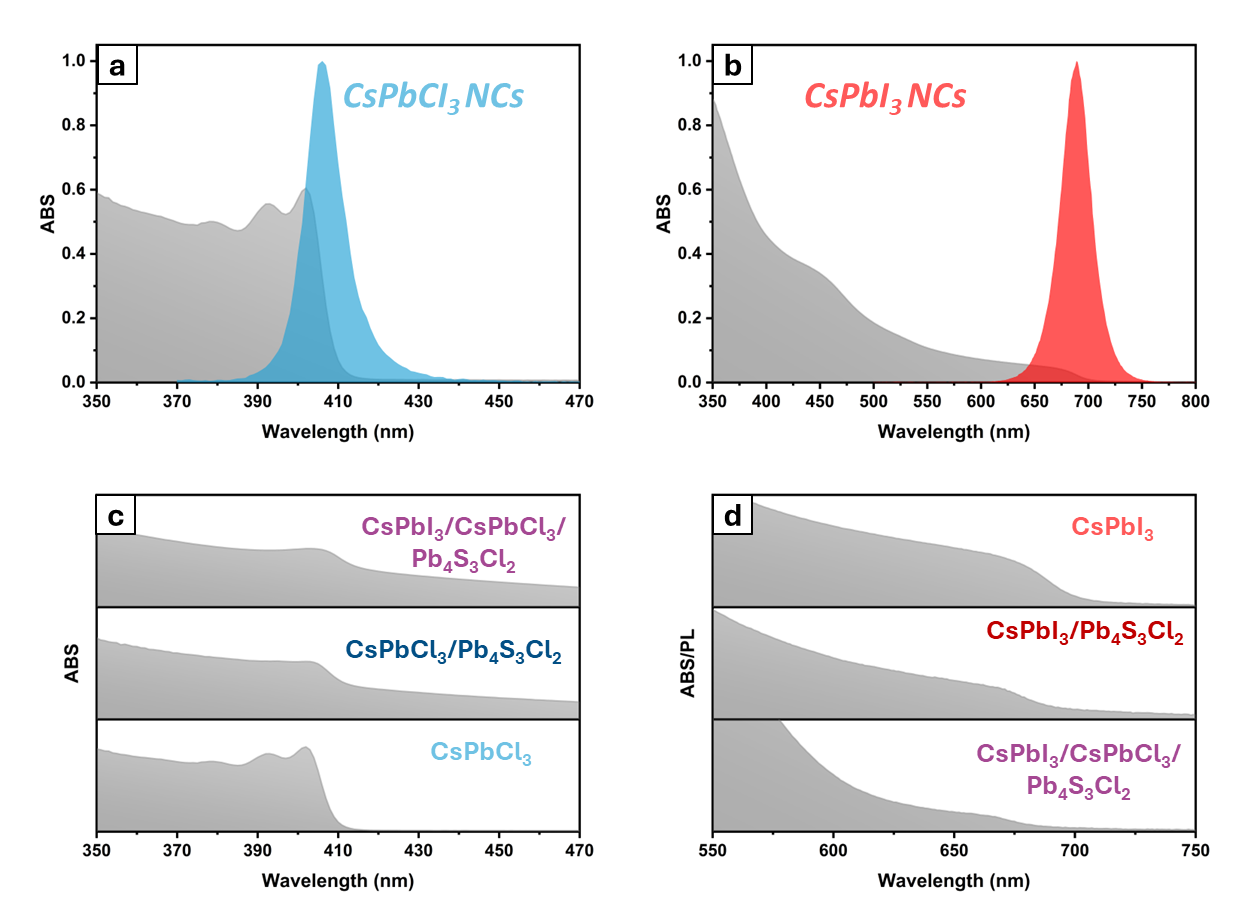


**Figure S10.** Optical absorption (grey) and PL (colored) spectra of as-synthesized pure (a) CsPbCl_3_ and (b) CsPbI_3_ NCs. (c) Comparison of the optical absorption spectra between free-standing CsPbCl_3_ NC, CsPbCl_3_-Pb_4_S_3_Cl_2,_ and partially exchanged CsPbI_3_-CsPbCl_3_-Pb_4_S_3_Cl_2_ heterostructures. (d) Comparison of the optical absorption spectra between free standing CsPbI_3_ NC, partially exchanged CsPbI_3_-CsPbCl_3_-Pb_4_S_3_Cl_2_, and fully exchanged CsPbI_3_-Pb_4_S_3_Cl_2_ heterostructures.

**
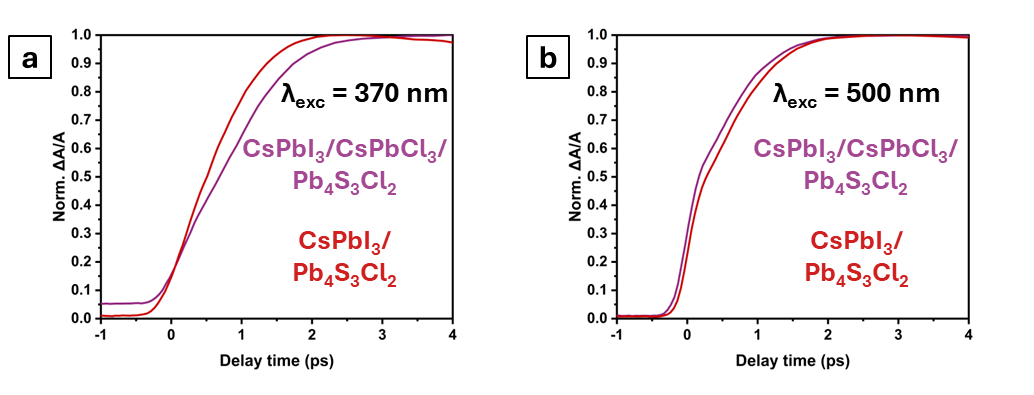
**

**Figure S11.** Transient absorption rise profile of the CsPbI_3_ exciton (ground-state bleaching,GSB) under (a) 370 nm excitation and (b) 500 nm excitation.

**
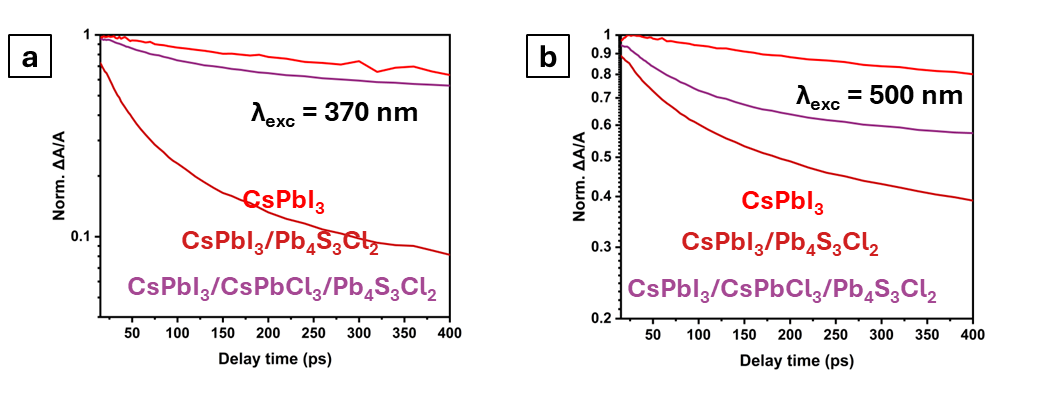
**

**Figure S12.** Transient absorption decay profile of the CsPbI_3_ exciton GSB under (a) 370 nm excitation and (b) 500 nm excitation.


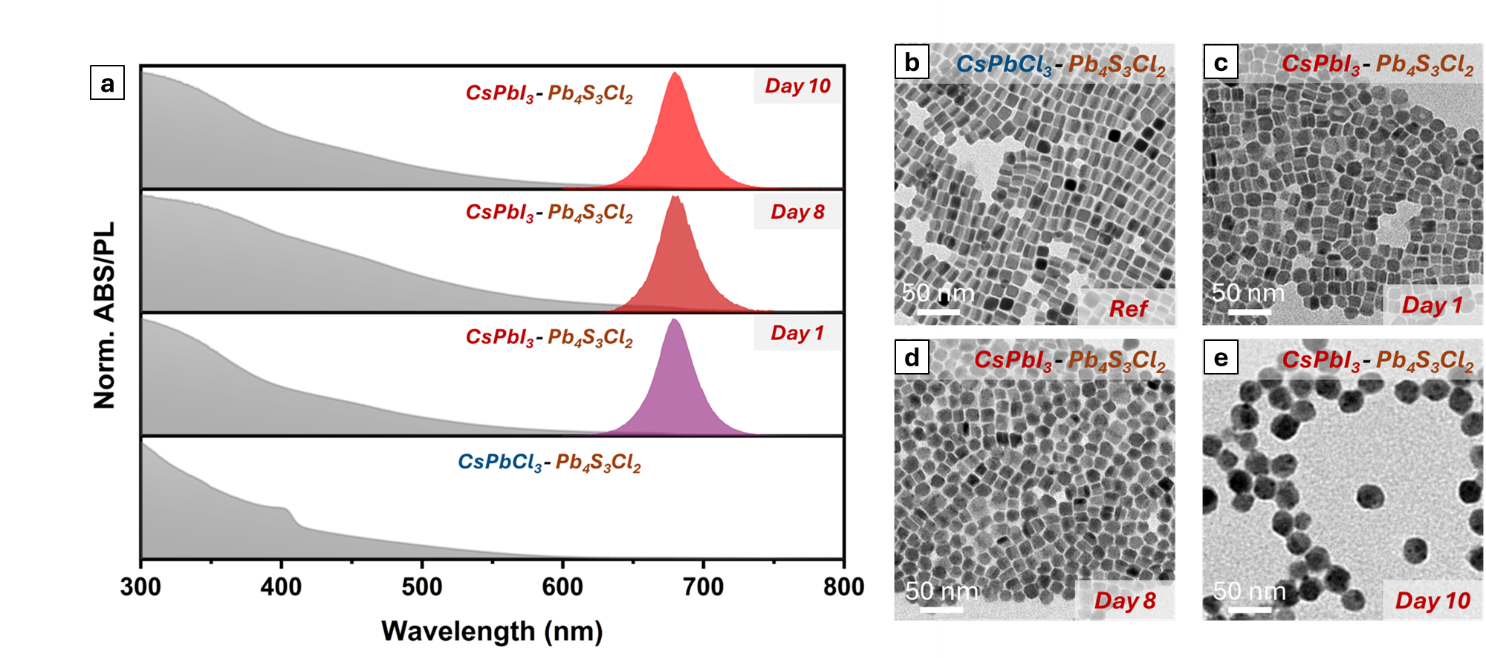


**Figure S13.** Steady-state optical and electron microscopy analyses of the initial CsPbCl₃–Pb₄S₃Cl₂ and fully exchanged CsPbI₃–Pb₄S₃Cl₂ NC heterostructures. (a) Optical absorption (grey) and PL (colored) spectra of the pristine and fully exchanged heterostructures measured after 1, 8, and 10 days of halide exchange reaction. TEM images of (b) pristine heterostructures and fully exchanged heterostructures after (c) 1 day, (d) 8 days, and (e) 10 days of reaction.
